# ROS‐Responsive Microneedle Patches Enable Peri‐Lacrimal Gland Therapeutic Administration for Long‐Acting Therapy of Sjögren's Syndrome‐Related Dry Eye

**DOI:** 10.1002/advs.202409562

**Published:** 2025-01-10

**Authors:** Jingqing Mu, Xiangyu Ding, Yapeng Song, Baoyue Mi, Xiaolong Fang, Baihua Chen, Bin Yao, Xuguang Sun, Xiaoyong Yuan, Shutao Guo, Xia Hua

**Affiliations:** ^1^ Aier Eye Hospital Tianjin University Fukang Road Tianjin 300110 China; ^2^ Changsha Aier Eye Hospital Changsha Hunan 410015 China; ^3^ Aier Eye Institute Changsha Hunan 410009 China; ^4^ Aier Academy of Ophthalmology Central South University Changsha Hunan 410125 China; ^5^ Key Laboratory of Functional Polymer Materials of Ministry of Education State Key Laboratory of Medicinal Chemical Biology Frontiers Science Center for New Organic Matter College of Chemistry Nankai University Tianjin 300071 China; ^6^ Department of Ophthalmology The Second Xiangya Hospital Central South University Changsha Hunan 410011 China; ^7^ Academy of Medical Engineering & Translational Medicine, Medical College Tianjin University Tianjin 300072 China; ^8^ Beijing Institute of Ophthalmology Beijing Tongren Eye Center Beijing Key Laboratory of Ophthalmology and Visual Sciences Beijing Tongren Hospital Capital Medical University Beijing 100005 China; ^9^ Tianjin Key Laboratory of Ophthalmology and Visual Science Tianjin Eye Institute Tianjin Eye Hospital Tianjin 300020 China

**Keywords:** drug delivery, dry eye, immunosuppression, microneedle, sustained release

## Abstract

Sjögren's syndrome‐related dry eye (SSDE) is a severe dry eye subtype characterized by significant immune cell attacks on the lacrimal gland. However, delivering immunosuppressive drugs to the lacrimal glands for SSDE therapy safely and sustainably poses significant challenges in clinical practice. Herein, a ROS‐responsive microneedle patch with detachable functionality (CE‐MN) is developed to enable straightforward and minimally invasive administration to the lacrimal gland area by penetrating the periocular skin. CE‐MN is loaded with immunosuppressive cyclosporin A and anti‐inflammatory drug epigallocatechin gallate, the latter also serving as a cross‐linker for the microneedle matrix. Poly(N‐isopropylacrylamide‐co‐butylacrylate), a temperature‐sensitive polymer is utilized to fabricate separable layers that allow controlled detachment of the base from the needle, reducing patient discomfort. CE‐MN is capable of modulating drug release by responding to ROS, facilitating on‐demand release, and drug accumulation to the lacrimal gland. Compared to traditional eye drops, the CE‐MN patch facilitated long‐acting drug delivery to the lacrimal gland for more than 48 h, demonstrating potent anti‐inflammatory and immunosuppressive effects in an SSDE mouse model by scavenging ROS and inhibiting the proliferation of Th1, Th17 cells, and macrophages. Overall, this long‐acting microneedle patch potentially offers a novel clinical approach for treating SSDE and other ocular chronic diseases.

## Graphical abstract

A new strategy for constructing smart gel‐based MN patches is developed to overcome the insufficient drug delivery of eye drops to the lacrimal gland. The gel‐based MN patches achieved long‐acting drug release in a ROS‐responsive manner, and separable base in a temperature‐responsive manner. The MN patches exhibited superior efficacy to eye drops in dry‐eye mice.

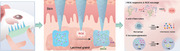

## 1 Introduction

Sjögren's syndrome‐related dry eye (SSDE) is a particularly severe and progressive subtype of dry eye, exceeding the severity of other causes.^[^ ^1^ ^]^ Sjögren's syndrome, which predominantly affects women, is a chronic autoimmune disease that primarily targets the exocrine, resulting in damage to the lacrimal and salivary glands.^[^ ^2^ ^]^ In individuals with Sjögren's syndrome, lymphocytic infiltration of the lacrimal gland forms organized foci, impairing glandular functionality and diminishing tear secretion, leading to the diverse clinical manifestations of dry eye.^[^ ^3^ ^]^ Meanwhile, an increased oxidative stress status, especially elevated reactive oxygen species (ROS), has been described in patients with Sjögren's syndrome.^[^ ^4^ ^]^ Oxidative stress and inflammation are in a causal cycle that exacerbates disease progression.

Cyclosporin A (CsA), an immunosuppressive agent, is the standard therapy in SSDE treatments.^[^ ^5^ ^]^ Clinically, eye drops and ointments, such as Restasis, Ikervis, and Cequa, are widely applied for treating SSDE;^[^ ^6^ ^]^ however, their efficacy is constrained by the tear and corneal barriers, with less than 5% of the drug being absorbed into ocular tissues, and an even smaller fraction reaching the lacrimal gland.^[^ ^7^ ^]^ Consequently, high dosages with repetitive applications are often required, potentially leading to systemic side effects and diminished patient adherence.^[^ ^8^ ^]^ Systemic administration of CsA is associated with significant adverse effects, such as an increased risk of infections and tumors, gastrointestinal disturbances, and nephrotoxicity.^[^ ^9^ ^]^ Furthermore, the persistent inflammatory response can lead to glandular atrophy, complicating the enrichment of drugs within the lacrimal gland. As a result, systemic treatment of SSDE is rarely employed in clinical practice due to these concerns.^[^ ^10^ ^]^ Thus, long‐acting CsA‐based therapies that can be administered locally for managing SSDE are highly desired.

Local injections can augment drug bioavailability, circumventing the barriers associated with topical and systemic administration, and may provide a sustained release profile.^[^ ^11^ ^]^ However, long‐acting formulations are usually formulated using implants or microspheres.^[^ ^12^ ^]^ These formulations are typically administered via injection or surgical intervention and are commonly utilized for the postoperative anti‐inflammatory treatment of ophthalmic diseases.^[^ ^13^ ^]^ In cases of ocular surface diseases that do not necessitate surgical intervention, this method of drug delivery not only induces discomfort but also elevates the risk of infection and the financial burden on patients.

Microneedles (MNs) are attractive, minimally invasive devices that effectively penetrate the stratum corneum of the skin, enhancing drug penetration and achieving long‐acting drug delivery for chronic diseases, such as diabetes, osteoporosis, gout, neurological, and cardiovascular conditions.^[^ ^14^ ^]^ The lacrimal gland is an epithelial tissue located in the lacrimal fossa above the outer orbit of the eye, with the eyelid skin being among the softest and thinnest in the body.^[^ ^15^ ^]^ Targeted drug delivery to the lacrimal gland using MNs by peri‐lacrimal gland administration is expected to improve precision, minimize invasiveness, and provide sustained drug release.^[^ ^16^ ^]^ More importantly, gel‐based MNs crafted from pathological stimuli‐responsive materials are capable of modulating drug release rates in accordance with disease severity, potentially avoiding the burst release and short effective duration associated with local injections.^[^ ^17^ ^]^

**Scheme 1:**
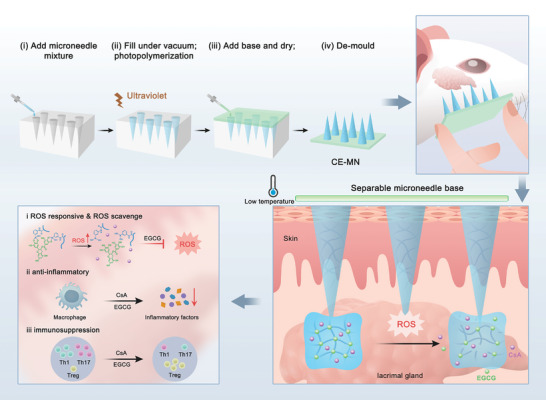
Schematic illustration of the fabrication process and therapeutic mechanism of the ROS‐responsive separable MN patches for SSDE therapy. The CE‐MN was fabricated from a PDMS mould using a two‐step casting process with in‐situ photopolymerization. The MN can painlessly penetrate the epidermis and are separated from the supporting base to stay close to the lacrimal gland, where the release of CsA and EGCG is triggered by H_2_O_2_ in the inflammatory microenvironment. As a result, EGCG reduces inflammation by scavenging ROS and inhibiting macrophages, and CsA modulates the immune environment by inhibiting the proliferation of Th1, Th17, and macrophages.

Here, we developed a ROS‐responsive MN patch loaded with CsA and (‐)‐epigallocatechin gallate (EGCG) with distinct solubility profiles to enable peri‐lacrimal gland administration and sustained ROS‐triggered drug delivery for SSDE treatment (**Scheme** **1**). In the MN patch, EGCG, an anti‐inflammatory and antioxidant abundant in green tea, served the dual role of an active therapeutic agent and a crosslinking agent, enabling the formation of ROS‐sensitive covalent borate bonds within the MN matrix. Furthermore, MNs were constructed from a polymer matrix made from N‐vinyl‐2‐pyrrolidone (NVP), a low‐viscosity liquid monomer. To ameliorate discomfort following administration, a temperature‐sensitive detachable base fabricated from poly(N‐isopropylacrylamide‐co‐butylacrylate) (PNIPAM‐B) was integrated to facilitate cold temperature‐induced separation from the needle body.^[^ ^18^ ^]^ Within the inflammatory microenvironment of SSDE, the heightened ROS levels catalyzed the cleavage of borate ester linkages, triggering a smart release of CsA and EGCG. Compared to traditional eye drops, the CE‐MN patch facilitated long‐acting drug delivery to the lacrimal gland for more than 48 h. Employing an SSDE dry eye mouse model, we demonstrated that this topical MN patch offered superior therapeutic efficacy over conventional eye drop formulations. This minimally invasive ocular drug delivery system may pave the way for novel lacrimal gland‐targeted therapeutic strategies for SSDE and long‐term topical delivery systems for other ocular diseases.

## 2 Result and Discussion

### 2.1 Preparation and Characterization of the CE‐MN Patch

Many gel‐based MNs have been reported for the delivery of small molecule drugs and proteins (such as dexamethasone sodium phosphate and CCL22) using water‐soluble polymers such as hyaluronic acid,^[^ ^19^ ^]^ gelatin,^[^ ^20^ ^]^ and polyvinyl alcohol,^[^ ^21^ ^]^ while the loading of hydrophobic drugs remains a challenge. In addition, these polymers are generally very viscous, which is not conducive to needle filling. Given that 40% of approved drugs and ≈90% of drugs in development are insoluble,^[^ ^22^ ^]^ it is imperative to develop new MNs that can deliver hydrophobic drugs and are easy to fill. NVP, a widely used monomer with low viscosity, was selected as the primary monomer due to its ability to effectively dissolve both hydrophobic CsA and hydrophilic EGCG. Subsequently, the UV photopolymerization method was employed to accomplish the polymerization of NVP to polyvinyl pyrrolidone. To fabricate ROS‐responsive MN, an EGCG‐based NVP matrix was first synthesized. 3‐(Acrylamido) phenylboronic acid (3APBA) and EGCG could form ROS‐responsive complexes (E‐A complex) solution through esterification reactions between catechol and phenylboronic acid (Figure S1, Supporting Information). Alizarin red S (ARS), a fluorescent indicator with a catechol structure, exhibits a pronounced alteration in fluorescence intensity upon binding with boric acid.^[^ ^23^ ^]^ Given its structural similarity to EGCG, ARS was used as a proxy to validate the reaction between the catechol group and 3APBA in the NVP solution. Figure S2 (Supporting Information) demonstrated a notable enhancement in fluorescence intensity at 570 nm following the mixing of ARS and 3APBA solutions for 2 h, signifying the formation of boronate esters. The reaction kinetics were monitored using a fluorescence spectrophotometer, as depicted in Figure S3 (Supporting Information), where an initial rise in fluorescence intensity plateaued after 2 h, indicating the reaction's completion. Subsequently, to ascertain the binding affinity of 3APBA for EGCG within the NVP solution, varying quantities of EGCG were introduced into a mixture containing both 3APBA and ARS. The fluorescence intensity at 560 nm was measured after a 3‐hour incubation period. Figure S4 (Supporting Information) illustrates a progressive reduction in fluorescence intensity with an increasing concentration of EGCG, indicative of E‐A complex formation in NVP, thereby attenuating the binding of 3APBA to ARS.

**Figure 1:**
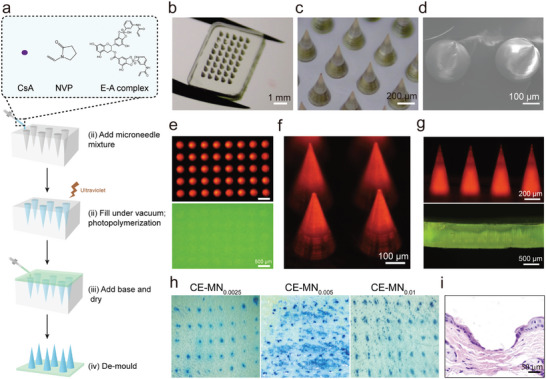
Characterization of the CE‐MN patch. a) Schematic illustration of the fabrication procedure of the CE‐MN patch from a PDMS mould using an in‐situ photopolymerization strategy. b,c) Typical digital photographs, and d) SEM images of CE‐MN. e) Fluorescence images of rhodamine‐B and FITC‐loaded MN for top, f) front, and g) side views. h) Digital images of mice skin stained with trypan blue after three types of MN pressing. i) Typical H&E images of mice skin after MN pressing.

Then, the two‐step casting procedure was used to fabricate the CsA‐loaded detachable MN patch (CE‐MN, **Figure** **1**a). The needles were formed via photopolymerization of a mixed solution containing the previously described E‐A complex‐based NVP matrix, CsA, and photoinitiator Irgacure 2959. This solution was cast into a polydimethylsiloxane (PDMS) mold to create the MN array. To facilitate controlled detachment of the MNs from the base, poly(N‐isopropylacrylamide‐co‐butyl acrylate) (PNIPAM‐B), a thermally responsive polymer with a lower critical solution temperature of 14—16 °C, was selected as the primary matrix for the base. To enhance the mechanical strength of the base, polyvinyl alcohol (PVA) was incorporated into the PNIPAM‐B solution at a weight ratio of 1:1. The PNIPAM‐B/PVA mixture was poured over the MNs in the PDMS mold and allowed to set overnight in a dry oven at 35 °C. The CE‐MN patches were then carefully peeled away from the PDMS mold. As illustrated in Figures 1b,c and Figure S5 (Supporting Information), each CE‐MN patch features a 5 × 8 array of conical‐shaped MNs with a center‐to‐center distance of 500 µm between adjacent tips. The MNs have a height of ≈600 µm and a base radius of ≈150 µm. The morphology of the MNs was further characterized using scanning electron microscopy (SEM), which confirmed their uniform morphology and geometrical consistency (Figure 1d).

It is inferred that the E‐A complex has a significant influence on the mechanical strength and drug release performance of CE‐MN due to its serving as a ROS‐sensitive cross‐linking agent. Thus, we explored a range of molar ratios of the E‐A complex to NVP, spanning from 0.0025 to 0.02. Upon incrementing the ratio to 0.02, we encountered an excessively adhesive needle matrix, which impeded the complete removal of the needles from the mold. Therefore, the molar ratio range of 0.0025–0.01 was chosen for further experimentation. Subsequently, to ascertain the independence between the needle matrix and the base, rhodamine B was introduced into the needle matrix, while the base was fabricated with a fluorescein isothiocyanate isomer I (FITC)‐doped PNIPAM‐B/PVA solution. As depicted in Figures 1e–g, rhodamine B fluorescence was localized exclusively within the needle, with the PNIPAM‐B/PVA matrix forming a uniform layer in apposition to the needle. Furthermore, the MN patch's ability to penetrate skin was evaluated using ex vivo mouse skin. Figure 1h illustrates that all CE‐MN formulations effectively breached the stratum corneum, as evidenced by the aggregation of trypan blue within the micropores. This finding was corroborated by hematoxylin and eosin (H&E) staining (Figure 1i; Figures S6, S7, Supporting Information), which revealed distinct notches of post‐microneedle penetration in mice and porcine skin. Following the application of the patch to the skin, the base was successfully detached by reducing the skin temperature to ≈10 °C, as shown in Figure S8 (Supporting Information). In summary, we successfully developed an MN patch with a detachable function, demonstrating the capacity to penetrate the skin barrier with its rigid needles.

### 2.2 ROS Sensitivity and Biosafety of MN System

**Figure 2:**
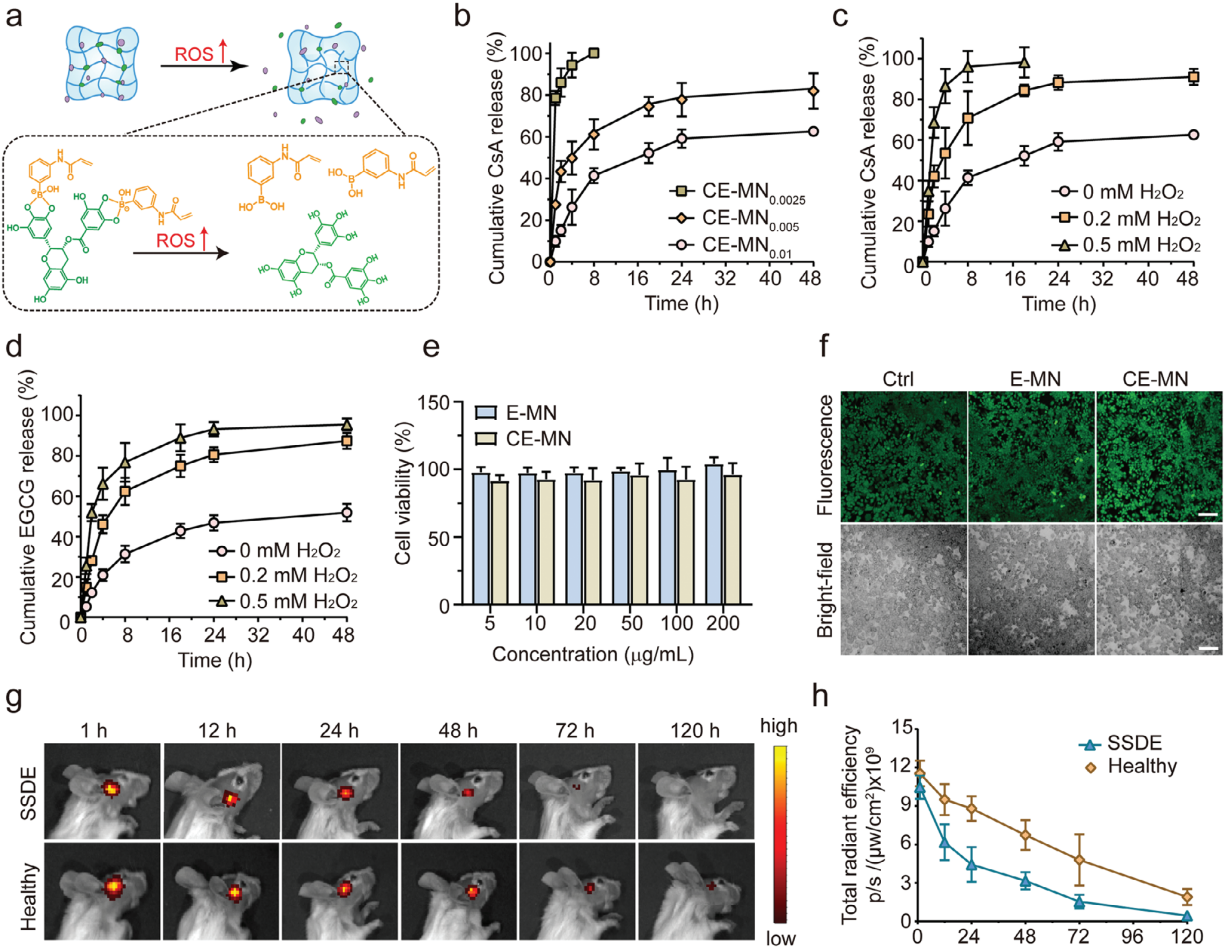
Characterization of the drug release profiles and biosafety of CE‐MN. a) Illustration of the mechanism of ROS‐triggered drug release from MNs. Upon exposure to high levels of ROS, borate ester bonds are cleaved, promoting the release of CsA and free EGCG from MNs. b) Cumulative in vitro release of CsA from three kinds of CE‐MN systems at phosphate buffer (pH 7.4) (*n* = 3, mean ± SD). (c, d) Cumulative in vitro release of (c) CsA and (d) EGCG from the CE‐MN_0.01_ system at different concentrations of H_2_O_2_ (*n* = 3, mean ± SD). e) Cell viability of NIH3T3 cells after co‐culture with E‐MN and CE‐MN for 24 h (*n* = 3, mean ± SD). f) Live/dead staining fluorescence images of NIH3T3 cells after co‐culturing with E‐MN and CE‐MN for 24 h. Green fluorescence: Calcein AM; red fluorescence: PI. g) Representative in vivo fluorescence images at different time points (1, 12, 24, 48, 72, and 120 h) after insertion of CE‐MN patches into ConA‐induced SSDE mice or healthy mice. h) Quantitative analysis of fluorescence intensities in the periocular region according to the in vivo images of mice (*n* = 3, mean ± SD).

Given that borate ester bonds are dynamic and can readily form and cleave under mild conditions, we explored the hypothesis that the CE‐MN could respond to ROS to facilitate controlled drug release (as illustrated in **Figure** **2**a). Our initial investigation involved examining the release kinetics of CsA from MNs with varying cross‐linking ratios in phosphate buffer (pH 7.4) (Figure S9, Supporting Information). Figure 2b and Figure S10 (Supporting Information) depicted a relatively rapid release profile for CE‐MN_0.0025_, with the release of CsA nearing completion within 8 h. An increase in the cross‐linking agent's proportion corresponded with a gradual decrease in the CsA release rate from the MNs. After 48 h, the cumulative release from CE‐MN_0.005_ reached 81.9%. Furthermore, the release of CsA from CE‐MN_0.01_ reached only 62.6% within the same timeframe, which was slower compared to other formulations and aligned with the desired long‐acting release profile. Consequently, CE‐MN_0.01_ was chosen for subsequent experiments and will hereafter be referred to as CE‐MN in the text. To simulate a high ROS environment, H_2_O_2_ was introduced to the phosphate buffer. The release rate of CsA from CE‐MN incubated in phosphate buffer with 0.2 mm H_2_O_2_ significantly increased, resulting in 91.1% of CsA being released within 48 h. The release rate of CsA escalated with rising H_2_O_2_ concentrations, as shown in Figure 2c and Figure S11 (Supporting Information). Additionally, we assessed the release kinetics of EGCG from CE‐MN using the Folin & Ciocalteu's phenol reagent method. Consistent with the CsA results, EGCG demonstrated a more rapid release in the presence of H_2_O_2_ in phosphate buffer (as shown in Figure 2d). These findings indicate that CE‐MN exhibits a high degree of sensitivity to the ROS‐rich microenvironment and is expected to achieve pathological stimulus‐triggered release in vivo.

Before cellular experimentation, the cytocompatibility of the CE‐MN was evaluated by employing the cell counting kit‐8 assay (CCK‐8) and Live/Dead staining. The E‐MN was fabricated following the same protocol as the CE‐MN, with the exclusion of CsA incorporation. Mouse embryonic fibroblast cells (NIH3T3) and human corneal epithelial cells (HCEC) were cultured in media containing varying concentrations of MNs for a duration of 24 h at a temperature of 37 °C. Subsequently, the CCK‐8 assay was conducted to assess cellular activity. The findings indicated that the viability of NIH3T3 cells remained above 96.1% after a 24 h exposure to E‐MN or CE‐MN, even at the highest MN concentration of 200 µg mL^−1^, as depicted in Figure 2e. Analogous outcomes were observed in the cytotoxicity assays conducted on HCEC cells, as illustrated in Figure S12 (Supporting Information), which underscored the excellent biocompatibility of the MNs. Thereafter, Live/Dead staining was conducted to visually assess cell cytocompatibility, where green and red fluorescence signify live and dead cells, respectively. It was observed that the majority of both NIH3T3 and HCEC cells maintained a normal morphology. Cells exposed to a concentration of 200 µg mL^−1^ of E‐MN or CE‐MN demonstrated a proliferation trend comparable to that of the control group, as shown in Figure 2f and Figure S13 (Supporting Information). Consequently, the MNs have exhibited satisfactory biocompatibility, suggesting their suitability for further in vivo applications.

Motivated by the outcomes of the in vitro experiments, we proceeded to assess the in vivo ROS‐responsive behavior of the CE‐MN patch. The mouse model for SSDE was induced through topical administration of SSDE (ConA), known to stimulate T cells, rapidly induce substantial T cell infiltration, and elevate ROS levels in local tissues, thereby replicating the clinical symptoms associated with SSDE.^[^ ^24^ ^]^ Cy5‐labeled CE‐MNs were applied to the SSDE or healthy mice, followed by monitoring using the IVIS Animal Imaging system (Figures 2g,h). After treatment, the fluorescence signals from normal mice were kept for 120 h. In contrast, the fluorescence signals in SSDE mice were nearly undetectable after 72 h. These results verified the smart responsive release profile of CE‐MNs in vivo, indicating that CE‐MN could release drugs sustainably under normal physiological conditions and accelerate drug release in inflammatory environments. To further evaluate the impact of ROS‐responsiveness on drug enrichment within the lacrimal gland, we determined the concentration of EGCG and CsA in the lacrimal glands of SSDE or healthy mice at various time points following the administration of the CE‐MN patch. As depicted in Figure S14 (Supporting Information), the accumulation of CsA and EGCG in the lacrimal gland of SSDE mice was markedly greater than that in healthy mice, suggesting that the drug release from CE‐MN was significantly accelerated in an environment with elevated ROS levels. Furthermore, the time to reach peak drug concentration (*T_max_*) for CsA was found to be shorter than that for EGCG, which might be attributed to the fact that CsA was physically encapsulated in MNs while EGCG was crosslinked as MN skeleton. These results indicated that the as‐prepared CE‐MN had an in vivo ROS‐responsive release behavior.

### 2.3 In Vitro ROS‐Scavenging and Anti‐Inflammatory Activities of CE‐MN

**Figure 3:**
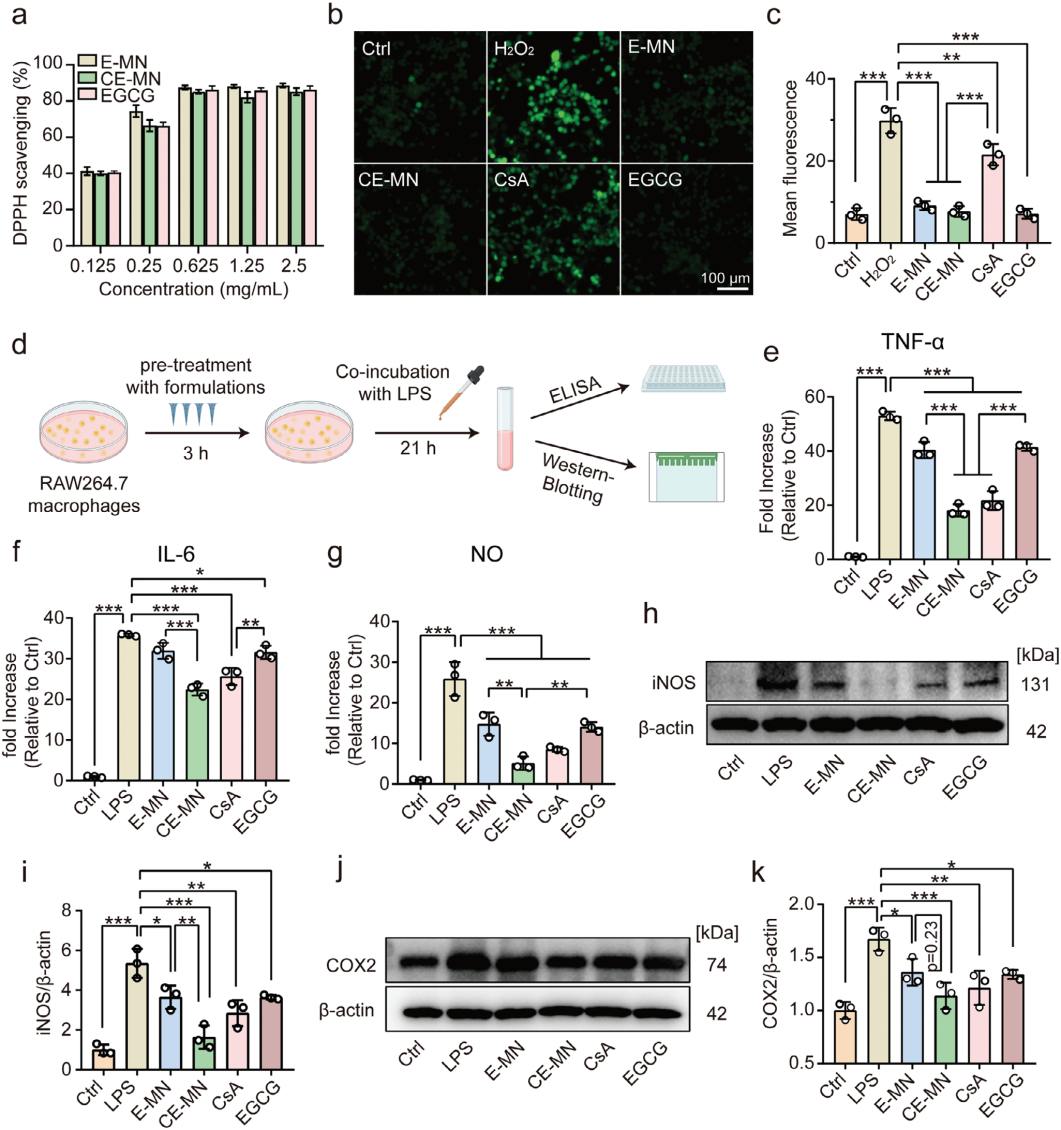
ROS‐scavenging capabilities and anti‐inflammation effect of CE‐MN. a) DPPH scavenging capabilities by the E‐MN, CE‐MN, and EGCG in different concentrations (*n* = 3, mean ± SD). b, c) Representative fluorescence images and statistical analysis of NIH3T3 after different treatments via DCFH‐DA staining (*n* = 3, mean ± SD). d) Scheme of the experimental procedures to assess the inflammation level in RAW264.7 cells. Expression level of e) TNF‐α, f) IL‐6, and g) NO in LPS‐induced RAW264.7 cells treated with different formulations for 24 h (*n* = 3, mean ± SD). Western blotting images of h) iNOS and j) COX2 protein expressions in LPS‐induced RAW264.7 cells treated with different formulations for 24 h. Densitometry data of i) iNOS and k) COX2 were generated from the Western blot images (*n* = 3, mean ± SD). ^*^p < 0.05, ^**^p < 0.01, ^***^p < *0.001* (one‐way ANOVA and Tukey's multiple comparison tests).

In SSDE, lacrimal gland cells are persistently subjected to ROS, which promotes the production of inflammatory cytokines.^[^ ^25^ ^]^ Therefore, the capacity of MNs to eliminate ROS was investigated. The in vitro ROS‐scavenging activity of MN was assessed using the DPPH free radical scavenging assay (**Figure** **3**a). This finding indicated that both the E‐MN and CE‐MN possessed ROS‐scavenging effects that were equivalent to those of free EGCG at corresponding concentrations. When the concentration exceeded 0.625 mg mL^−1^, both E‐MN and CE‐MN exhibited obvious oxidation resistance with over 85% radical scavenging efficiency, suggesting excellent radical scavenging performance. Moreover, the DPPH scavenging performance of MNs was observed to be concentration‐dependent. To further evaluate the intracellular ROS‐scavenging capacity of the MNs, E‐MN, CE‐MN, or free drugs were co‐incubated with NIH3T3 cells treated with H_2_O_2_, a stimulus that induces excessive ROS production in cells. Thereafter, intracellular ROS levels were quantified using the DCFH‐DA assay kit. Figures 3b,c illustrated a notable reduction in fluorescence in NIH3T3 cells treated with E‐MN, CE‐MN, or free EGCG, with fluorescence intensities resembling those of the control group. In contrast, cells treated with free CsA displayed intense fluorescence, with an intensity akin to that of the positive control group treated exclusively with H_2_O_2_. These observations suggest that EGCG encapsulated within the MNs plays a pivotal role in augmenting the ROS‐scavenging efficacy.

Macrophages serve as key effector cells in the inflammatory and oxidative stress pathways associated with the pathogenesis of SSDE. They are recruited in large numbers and activated after CD4^+^ T cell infiltration, where they exacerbate and sustain inflammation in SSDE.^[^ ^26^ ^]^ To elucidate the anti‐inflammatory effects of CE‐MN on macrophages, we utilized lipopolysaccharide (LPS) to induce inflammation in mouse monocyte macrophage leukemia (RAW 264.7) cells. As shown in the scheme of experimental procedures (Figure 3d), RAW264.7 cells were pretreated with formulations for 3 h, then stimulated with LPS for 21 h, and finally were evaluated by enzyme‐linked immunosorbent assay (ELISA) kit and Western blotting. As shown in Figures 3e–g, the expression levels of TNF‐α, IL‐6, and NO in the supernatant of RAW 264.7 cells were significantly increased after LPS stimulation, indicating successful induction of the inflammation model. The expression of TNF‐α and NO decreased moderately after treatment of E‐MN and EGCG, suggesting effective inflammation inhibition by EGCG. It has been reported that EGCG exerts an anti‐inflammatory effect by promoting the polarization of macrophages from a pro‐inflammatory M1 phenotype to an anti‐inflammatory M2 phenotype, thereby reducing the release of inflammatory mediators.^[^ ^27^ ^]^ After free CsA treatment, the expression of three cytokines was markedly reduced, indicating the powerful anti‐inflammatory effects of CsA, which was consistent with previous reports.^[^ ^28^ ^]^ It is well known that the nuclear factor of activated T cells (NFAT) is the target of CsA to regulate T lymphocytes.^[^ ^5^ ^]^ However, NFAT is expressed not only in lymphocytes but also in intrinsic immune cells such as macrophages and neutrophils. Therefore, CsA can target not only lymphocytes but also innate immune cells such as monocytes/macrophages, dendritic cells, and neutrophils.^[^ ^29^ ^]^ Notably, the treatment with CE‐MN yielded the most pronounced reductions in cytokine expression levels, with a 65.8%, 37.4%, and 80.3% decrease in tumor TNF‐α, IL‐6, and NO expression, respectively. Given the elevated expression of inducible nitric oxide synthase (iNOS) and cyclooxygenase‐2 (COX‐2) in inflammation‐stimulated cells, we further evaluated the protein expression of these two markers in LPS‐induced RAW 264.7 cells treated with various formulations using Western blotting. As shown in Figures 3h–k, LPS induced a 5.3‐fold increase in iNOS and a 1.7‐fold increase in COX‐2 in RAW 264.7 cells. Among the treatment groups, the CE‐MN group demonstrated the most robust effects, with an 85.4% reduction in iNOS and a 79.5% reduction in COX‐2. Furthermore, to assess the efficacy of the CE‐MN as a therapeutic strategy, macrophages were stimulated with LPS and subsequently treated with various formulations. The inflammatory responses observed across different treatment groups were comparable to those in the pre‐treatment experiments, confirming the therapeutic potential of CE‐MN in modulating the inflammatory response (Figure S15, Supporting Information). Collectively, these findings indicated that CE‐MN, through the combined therapeutic action of CsA and EGCG, exhibited superior anti‐inflammatory effects and held significant potential for SSDE treatment.

### 2.4 In Vivo Therapeutic Effects of CE‐MN on the SSDE Model

**Figure 4:**
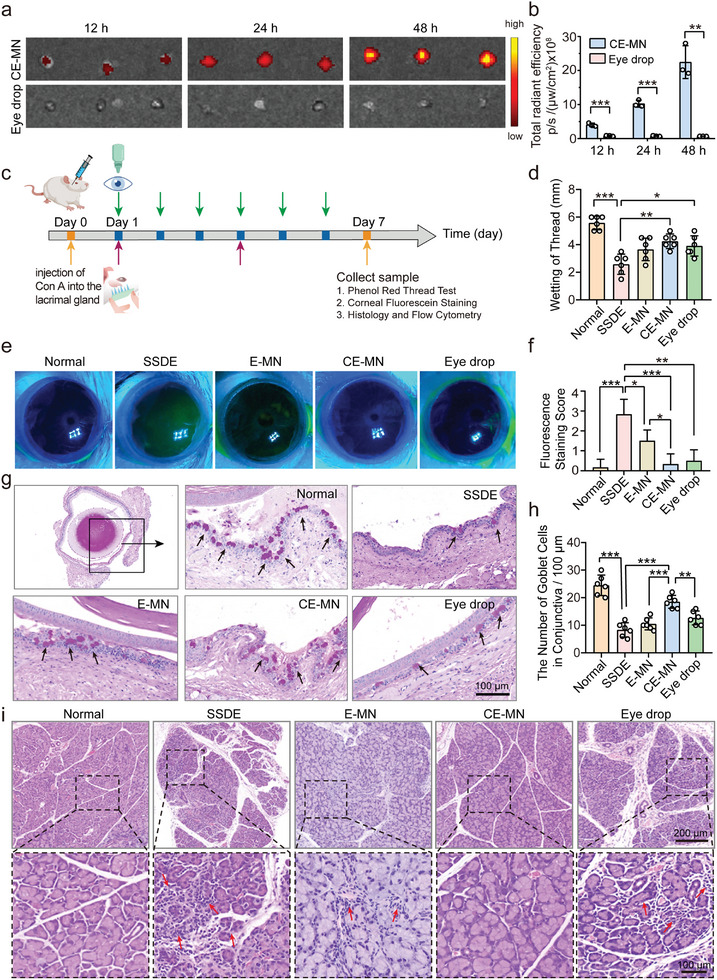
The therapeutic effect of various therapies on ConA‐induced dry eye models. a) Fluorescence images and b) corresponding fluorescence intensities of dissected lacrimal gland at 12, 24, and 48 h after insertion of CE‐MN patches or Eye drops in SSDE mice (*n* = 3, mean ± SD). c) Scheme of SSDE modeling and treatment. The model mice were established by injection of 10 µL ConA (10 mg mL^−1^) into each lacrimal gland. Then, the mice received E‐MN or CE‐MN patch on day 1 and day 4 (The dose of CsA and EGCG were 30 and 22.8 µg per mouse per side, respectively). The positive control group received a topical instillation of 10 µL Eye drops twice a day (The daily doses of CsA and EGCG were 10 and 7.6 µg per mouse per side, respectively). Therapeutic effects were recorded and evaluated on day 7. d) Wetted phenol red thread lengths for tear production evaluation (*n* = 6, mean ± SD). e) Representative images of fluorescein sodium staining and f) corresponding staining scores of the ocular surface on treated mice (*n* = 6, mean ± SD). Green staining on the cornea is a positive index of injury. g) Representative images of conjunctival PAS staining and h) corresponding statistical analysis of the number of goblet cells per 100 µm (*n* = 6, mean ± SD). Goblet cells are the purple cells located in the conjunctiva highlighted with black arrows. i) Representative H&E staining images of the lacrimal gland. Lymphocyte infiltration and structural disorder are labeled with red arrows. ^*^p < 0.05, ^**^p < 0.01, ^***^p < 0.001 (b, unpaired student's *t*‐test; d,f,h, one‐way ANOVA and Tukey's multiple comparison tests).

Initially, we investigated the positional distribution of the CE‐MN post‐administration by collecting tissue samples from the puncture site of the SSDE mice at 1 and 48 h following the application of Rhodamine B‐loaded MN. As shown in Figure S16 (Supporting Information), the red‐colored needle gel was distinctly visible within the subcutaneous tissue of the mice 1 h post‐administration, with a distance of ≈350–500 µm to the edge of the lacrimal gland. Over time, the intensity of the red color progressively diminished. To evaluate the comparative accumulation of drugs within the lacrimal gland following administration via MNs or clinically used eye drops, the SSDE mice were euthanized at 12, 24, and 48 h post‐treatment with either the Cy5‐loaded CE‐MN or the equivalent Eye drop formulation. Lacrimal glands were harvested for ex vivo imaging, as shown in **Figure** **4**a. The fluorescence signal was discernible and demonstrated a gradual increase in the lacrimal glands of mice administered the CE‐MN over the 48‐h period. In stark contrast, minimal fluorescence was detected following treatment with the Eye drop formulation within the same timeframe (Figure 4b). These observations suggested the superior targeting and drug delivery efficiency of the CE‐MN system to the lacrimal gland.

Inspired by the superior antioxidant ability, anti‐inflammatory, and delivery efficiency, we evaluated the therapeutic outcome of CE‐MN in a mouse model of SSDE. The SSDE mice were subjected to various treatments, including E‐MN, CE‐MN, or Eye drops (a mixture obtained by incorporating an appropriate amount of EGCG into Restasis). E‐MN and CE‐MN were applied on the 1st and 4th days, whereas the Eye drop was administered twice daily for a period of 6 days. Both normal mice and SSDE mice were used as control groups. The therapeutic effects were evaluated on the 7th day (Figure 4c). The Schirmer test, a conventional method for assessing dry eye severity through tear production measurement, was initially employed to evaluate dry eye symptoms (Figure 4d; Figure S17, Supporting Information). As shown, SSDE mice exhibited a severe deficit of tear secretion with a wetted length of 2.6 ± 0.7 mm, implying severe dry eye symptoms. The E‐MN group elevated tear secretion to some extent, which produced a wetted length of 3.7 ± 0.8 mm. Tear deficiency was significantly alleviated after the treatment with CE‐MN with the thread wetting of 4.3 ± 0.5 mm, which was higher than eye drop with a wetted length of 3.9 ± 0.7 mm. The integrity of corneal epithelium, as an indicator of disease severity, was evaluated by fluorescein staining (Figures 4e,f). No staining was observed in the corneas of the normal group, with a staining score of 0.17 ± 0.41, indicative of an intact corneal epithelium. Conversely, the staining score for the SSDE mice reached 2.83 ± 0.75. The staining score reduced to 1.50 ± 0.55 after treatment with E‐MN, implying the dry eye symptoms were relieved but not completely cured. After 7 days of treatment with CE‐MN, the corneal staining score decreased to 0.33 ± 0.52, which is lower than the eye drops scored 0.50 ± 0.55, demonstrating an outstanding therapeutic outcome of CE‐MN. Moreover, CE‐MN treatment significantly reduced the frequency of administration, which helped to improve patient compliance.

Goblet cells could lubricate the ocular surface by secreting mucins, and their absence is a key feature of dry eye. After one week, the eyeballs were excised, and the conjunctiva was stained with Periodic Acid Schiff (PAS) to distinguish goblet cells (displayed as bright purple/pink cells). As shown in Figures 4g,h, the goblet cells are abundant in the conjunctival fornix of the normal mice, reaching a density of 24.5 ± 3.7 cells/100 µm. On the contrary, the goblet cell density in disease mice was significantly reduced to 8.3 ± 2.6 cells/100 µm. These goblet cells were sparsely dispersed along the conjunctival epithelium, leading to mucin insufficiency and tear film instability. Notably, the density of goblet cells in the CE‐MN group recovered to 18.5 ± 2.3 cells/100 µm, exhibiting better therapeutic effects than the other groups. We speculated that CsA played a dominant role in increasing goblet cell density by effectively inhibiting the cytokines produced by T cells, which are known to induce goblet cell apoptosis. Consequently, CE‐MN showed the best therapeutic effect, primarily due to its ability to surmount the ocular surface barrier and achieve higher delivery efficiency compared to eye drops.

The lacrimal gland, the main site of tear secretion, is the main gland affected in patients with SSDE. Lacrimal glands were collected and stained with H&E to evaluate the degree of inflammation (Figure 4i). The acini in healthy mice are uniform in size, neatly arranged, and normal in morphology. After suffering from dry eye, numerous acini became heterogeneous in size and chaotically arranged, showing concrete manifestations of atrophy and fusion, along with vasodilation and lymphocytic infiltration. The acini morphologies and inflammation problem did not disappear after the administration of Eye drops, which made it difficult to deliver drugs to the lacrimal gland. After the treatment of E‐MN, the structure of the lacrimal gland was effectively restored, but the inflammatory cell infiltration remained. Surprisingly, treatment with CE‐MN markedly alleviated the structural disorder and inflammatory infiltration to a near‐normal state. All these results confirmed that CE‐MN can significantly restore the morphology and structure of acinar cells and goblet cells in the SSDE model, as well as the function of tear secretion.

Baudouin et al. introduced the concept of a “vicious cycle of inflammation” as a core driver in dry eye.^[^ ^30^ ^]^ This cycle includes tear film instability, tear hyperosmolarity, apoptosis of corneal/conjunctival cells, and inflammation in the ocular surface. The lacrimal gland, the main site of tear secretion, is crucial in maintaining tear film integrity.^[^ ^1^, ^31^ ^]^ Dysfunction of the lacrimal gland leads to a deficiency in the aqueous components of the ocular surface, triggering an inflammatory vicious cycle and consequently resulting in dry eye and the potential for significant surface pathology. Additionally, the lacrimal gland produces various proteins and products essential for the growth and maintenance of host tissue within the tear film. We employed MNs to deliver CsA and EGCG to the peri‐lacrimal gland directly, reducing inflammation and reactive oxygen species levels of the lacrimal gland. The secretion of tears increased, the vicious cycle of dry eyes was broken, and an overall improvement in ocular surface condition occurred. A similar phenomenon was reported. A Treg recruitment microsphere was injected into the lacrimal gland of dry‐eye mice, resulting in reduced inflammation of the lacrimal gland and improved ocular surfaces, such as tear clearance rate, corneal epithelial integrity, and goblet cell density.^[^ ^32^ ^]^

### 2.5 CE‐MN Inhibited Th1/Th17 Cells Response but Enhanced Treg Cells Response In Vivo

**Figure 5:**
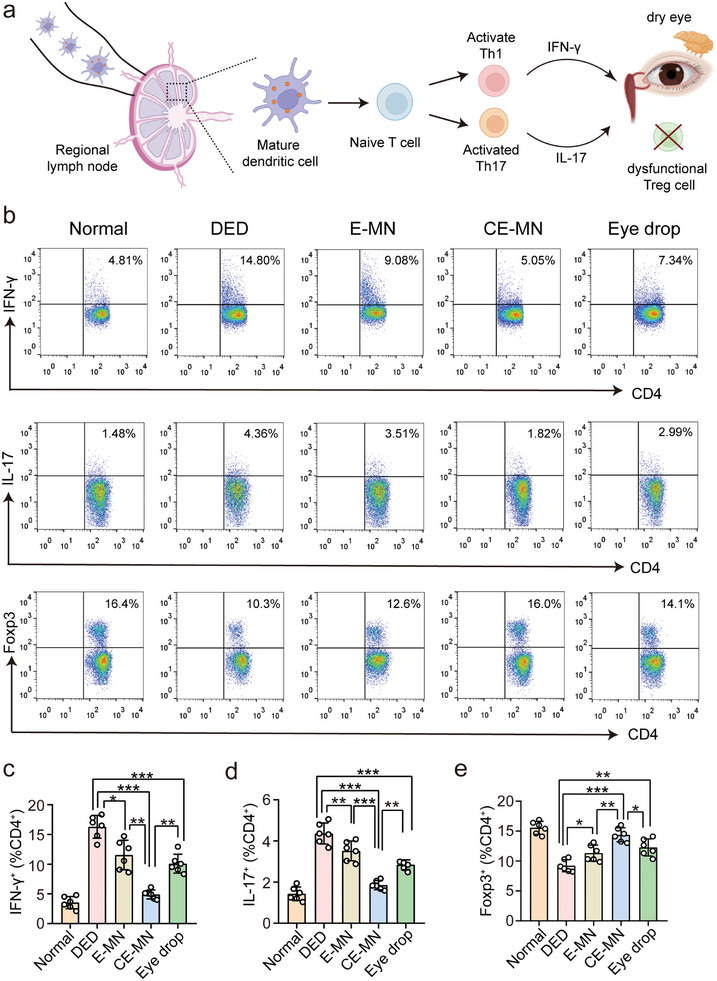
CE‐MN regulated Th1, Th17, and Treg responses in vivo. a) Schematic illustration of immune responses and a vicious cycle of inflammation in SSDE. b) Flow cytometric analysis of CD4^+^IFN‐γ^+^, CD4^+^IL‐17^+^, and CD4^+^Foxp3^+^ cells in cervical lymph nodes of normal mice or SSDE mice treated with saline, E‐MN, CE‐MN, or Eye drop. c–e) The quantitative results of CD4^+^IFN‐γ^+^, CD4^+^IL‐17^+^, and CD4^+^Foxp3^+^ T cells in cervical lymph nodes after different treatments. (*n* = 6, mean ± SD). ^*^p < 0.05, ^**^p < 0.01, ^***^p < *0.001* (one‐way ANOVA and Tukey's multiple comparison tests).

The superior therapeutic effects prompted us to excavate into the therapeutic mechanism of CE‐MN. Regional lymph nodes are crucial sites for inducing an ocular surface immune response. In SSDE patients, the dendritic cells are activated and migrated to regional lymph nodes. Mature dendritic cells prime naive T cells into CD4^+^ IFN‐γ^+^ (Th1) and CD4^+^ IL‐17^+^ (Th17) cells. Activated Th1 and Th17 cells migrate back to the eye where they secrete IFN‐γ and IL‐17, causing lacrimal gland damage, goblet cell loss, disruption of the corneal barrier, and activation of innate immunity, while CD4^+^ Foxp3^+^ (Tregs) become dysfunctional (**Figure** **5**a).^[^ ^3b^ ^]^ To determine whether CE‐MN treatment could regulate CD4^+^ T cell responses, the phenotypic expression of T cells in cervical lymph nodes was analyzed by flow cytometry on the 7th day post‐treatment. As shown in Figures 5b–e, the proportions of Th1 cells and Th17 cells in the cervical lymph nodes were significantly enhanced, whereas the proportions of Treg cells were significantly decreased after injecting ConA. These results revealed that the ConA‐induced inflammation triggered a strong activation of pro‐inflammatory T cells, accompanied by inhibition of Treg cells, similar to the previous reports.^[^ ^32^ ^]^ Interestingly, CE‐MN treatment correlated with a significant reduction in Th1‐type and Th17‐type cell proliferation compared to E‐MN and Eye drop treatment, confirming the therapeutic merit of MNs in inhibiting Th1 and Th17 cell responses. Meanwhile, a distinct increase in the frequency of Treg cells was observed in the CE‐MN group compared to the E‐MN and Eye drop groups. These results suggested that CE‐MN suppressed the immunological response by decreasing Th1 and Th17 cells and potentiating Tregs in the cervical lymph node of SSDE mice, probably due to its higher delivery efficiency of CsA and EGCG to lymph nodes and lacrimal glands than Eye drop.

### 2.6 CE‐MN Effectively Suppressed Inflammation and Altered the Effector T Cells/Treg Balance in the Lacrimal Gland

**Figure 6:**
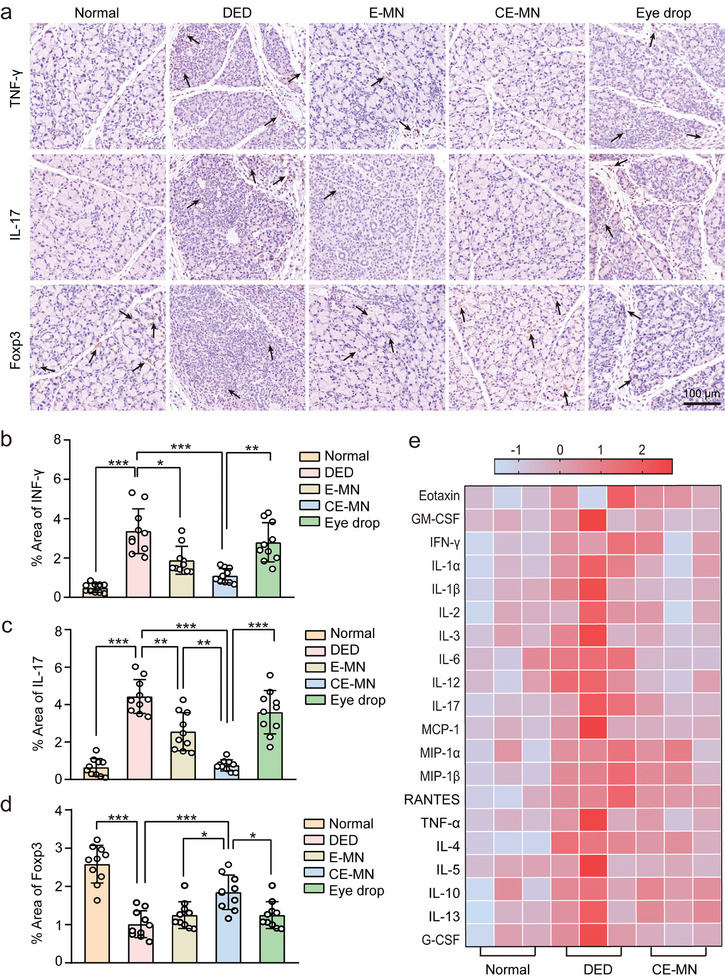
The anti‐inflammatory effect of CE‐MN in the lacrimal gland. a) Images and b‐d) quantification of the immunostaining for IFN‐γ, IL‐17 and Foxp3 on the lacrimal gland in different groups (*n* = 10, mean ± SD). The positive index was labeled with black arrows. e) Protein expression levels of 20 cytokines and chemokines in the lacrimal gland were detected by Luminex on day 7. ^*^p < 0.05, ^**^p < 0.01, ^***^p < *0.001* (one‐way ANOVA and Tukey's multiple comparison tests).

We further assessed the levels of IFN‐γ, IL‐17, and Foxp3 (a major immunosuppressive transcription factor of Treg) in the lacrimal gland by immunohistochemistry (**Figures** **6**a–d). Consistent with the flow cytometric analysis, a significantly increased expression of IFN‐γ and IL‐17 with a decreased infiltration of Foxp3 into the lacrimal gland was observed in the SSDE mice. E‐MN or Eye drop treatment resulted in a slight reduction in IFN‐γ and IL‐17 expression, while CE‐MN treatment exhibited an obvious reduction in IFN‐γ (67.4%) and IL‐17 (83.1%). In contrast, Foxp3 was significantly increased in the lacrimal glands of mice treated with CE‐MN compared to E‐MN and Eye drops. These results indicated that CE‐MN alleviated inflammation of the lacrimal gland by altering the equilibrium between effector T cells and regulatory T cells.

To precisely assess the inflammatory status of the lacrimal gland and to determine the capacity of CE‐MN to modulate the inflammatory milieu, SSDE mice were treated with and without CE‐MN, with normal mice serving as a control group. On day 7, we evaluated 20 common inflammatory cytokines at the protein level using a mouse cytokine array for the lacrimal gland tissues (Figure 6e). All data were normalized and graphically represented as a heat map. The SSDE group exhibited a markedly distinct cytokine expression profile, particularly for GM‐CSF, IFN‐γ, IL‐1α, IL‐1β, IL‐2, IL‐3, IL‐6, IL‐12, IL‐17, MCP‐1, and TNF‐α. At day 7 post‐treatment, CE‐MN efficaciously dampened the expression of pro‐inflammatory cytokines, including IFN‐γ, IL‐1α, IL‐2, IL‐6, and TNF‐α, with protein levels approximating those of the control group. These findings indicate that ConA‐induced immune activation led to a substantial release of pro‐inflammatory cytokines. However, CE‐MN treatment rapidly restored the inflammatory environment within the lacrimal gland, aligning with the results of immunohistochemical analysis.

To assess the systemic toxicity of the MNs, histological examination of major organs was conducted using H&E staining (Figure S18, Supporting Information). No obvious tissue damage or inflammation was observed in the treated groups, implying the biosafety of MN systems. Clinical treatment of SSDE patients is mainly based on increasing tear secretion, reducing inflammation, and inhibiting immune activation. However, eye drops are often ineffective because of the ocular surface barrier. Therefore, CE‐MN not only had a triple effect of ROS scavenging, anti‐inflammation, and immunosuppression but also achieved smart release and efficient drug delivery to the peri‐lacrimal gland based on the gel‐microneedle structure. To sum up, the CE‐MN system provided a long‐acting release of CsA and EGCG near the SSDE lesion site, which was a significant advancement over the conventional eye drop administration. Nevertheless, considering the physiological structural disparities between murine and humans, further research should focus on optimizing the MN patch design. This includes optimizing parameters such as needle length, size, and the dosage of drug loading to satisfy the clinical applicability of the MN patch.

## 3 Conclusion

The lacrimal gland constitutes an integral component of the lacrimal functional unit, yet its role in maintaining the homeostatic balance of the ocular surface has been underappreciated in the past. Traditional ophthalmic formulations encounter challenges due to the ocular surface barrier, which impedes the efficacious delivery of therapeutics to the lacrimal gland. MNs are of increasing interest in ophthalmology owing to their capacity to improve penetration, topical application, sustained release, minimal invasiveness, and potential for patient self‐administration. We have engineered a CsA‐loaded MN patch, crosslinked with the antioxidant EGCG, and integrated with a temperature‐sensitive detachable base. This innovative system is designed to address SSDE by effectively mitigating inflammation within the lacrimal gland and the ocular surface. CE‐MN exerted localized action on the peri‐lacrimal gland skin, exhibiting minimal invasiveness and excellent biosafety. Given the elevated pathological ROS levels in the lacrimal gland of patients with SSDE, the cross‐linked gel‐based MNs achieved long‐acting, on‐demand drug release in a ROS‐responsive manner for more than 48 h. CE‐MN could efficiently rescue the deterioration of SSDE by scavenging ROS, inhibiting inflammation, and altering the effector T cells/Treg balance. Comparatively, in an SSDE murine model, CE‐MN outperformed traditional eye drop formulations in immunosuppressive and anti‐inflammatory actions. The intelligent design and facile fabrication of these MNs pave the way for their application in the treatment of a broader spectrum of diseases.

## 4 Experimental Section

### The Preparation of the E‐A Complex

First, the E‐A complex was first prepared by dissolving 3APBA and EGCG in NVP. Briefly, 3APBA was dissolved in NVP at molar ratios of 1, 2, 4, and 8 mol%. EGCG was dissolved in NVP at molar ratios of 0.5, 1, 2, and 4 mol%. The EGCG solution was then added to the 3APBA solution at a molar ratio of 1:2 for EGCG:3APBA and mixed thoroughly for 30 s. The mixture was left at room temperature for 2 h to promote the formation of the E‐A complex (0.25, 0.5, 1, 2 mol% in NVP). ARS was used to verify the formation of phenylborate ester bonds in the NVP solution.^[^ ^33^ ^]^

### Fabrication and Characterization of MN Patches

The CE‐MN patch was prepared by an in situ polymerization process under UV light irradiation. The MN precursor solution was prepared by dissolving CsA (5.3 wt%) and Irgacure 2959 (1 mol%) in the E‐A mixture. The solution was deposited directly onto the PDMS mold surface. The mold was then held under a vacuum for 5 min to allow the solution to fill the needles of the MN mold. After removing the excess solution, the mold was exposed to a UV lamp (60 W; 365 nm) for 30 min. PNIPAM‐B powder was dissolved in deionized water (15 wt. %) and placed at 4 °C until completely dissolved; PVA was dissolved in deionized water (15 wt. %) and stirred overnight at 60 °C to dissolve completely. The two solutions were pre‐cooled in a cold room and mixed in the cold room to obtain the PNIPAM‐B/PVA solution (*v*/*v* = 1:1). The thermoresponsive MN matrix was prepared by adding the pre‐mixed PNIPAM‐B/PVA solution to the mold surface in the cold room. The mold was left to dry overnight in an oven at 35 °C. Finally, the MN patches were carefully peeled off the PDMS mold.

### SSDE Model Mice

Female BALB/c mice (7–8 weeks old) were purchased from Beijing Vital River Laboratory Animal Technology Co. Ltd. and raised under specific pathogen‐free conditions. All the animal protocols and guidelines were approved by the Institutional Animal Care and Use Committee of Tianjin University (Approval number: TJUE‐2022‐304). To induce SSDE, mice were anesthetized and then 10 mg mL^−1^ of concanavalin A (ConA) (Sigma–Aldrich, St. Louis, MO) in saline (10 µL) was injected into each lacrimal gland.

### Therapeutic Efficacy Assessment

The lacrimal gland of the mouse was injected with 10 µL ConA (10 mg mL^−1^) to induce the SSDE model. One day later, all mice were anaesthetized by intraperitoneal injection of 30 µL g^−1^ tribromethyl alcohol (#MA0478, Meilun) and randomly divided into five groups (normal, model, E‐MN, CE‐MN, and Eye drop). Eye drop was obtained by incorporating EGCG into Restasis (Allergan) to reach a concentration of 380 µg mL^−1^, which achieved the same ratio of CsA/EGCG as in CE‐MN. For MN treatment groups, MN patches were applied by peri‐lacrimal gland administration every 3 days from day 1 (The dose of CsA and EGCG is 30 and 22.8 µg per mouse per side, respectively). For Eye drop treatment groups, the mice received a topical instillation of 10 µL per eye twice per day (The content of CsA and EGCG is 5 and 3.8 µg per 10 µL Eye drop, respectively). Clinical assessments of dry eye, including tear secretion and ocular surface parameters, were utilized to assess the therapeutic efficacy on the 7th day. Schirmer test was measured using phenol red cotton threads (Jingming Co., Ltd, Tianjin, China). The thread was gently placed into the middle and outer 1/3 of the inferior conjunctival sac of the eye for 30 s and the wetting length was recorded in millimeters. Ocular fluorescein staining was performed to evaluate ocular surface parameters. 1 µL drop of 2% liquid fluorescein sodium was injected onto the ocular surface, followed by three artificial blinks. Corneal epithelial damage was examined under a cobalt blue light by a slit‐lamp microscope (SLM‐7E, KANGHUA, China). The cornea staining was scored by an ophthalmologist in a double‐blind fashion.

### Therapeutic Efficacy Assessment—Histopathology

The mice were sacrificed on the 7th day and the whole eyeball, lacrimal glands, and main organs (heart, liver, spleen, lung, kidney) were collected and fixed in 4% paraformaldehyde for hematoxylin‐eosin or PAS staining. To count goblet cells, three different PAS‐stained slides of each group were selected to observe the numbers of goblet cells in the superior and inferior conjunctival fornices. The lacrimal glands samples were sliced in 5 µm sections for INF‐γ (#BS‐0480R, Bioss), IL‐17 (#A00421‐3, Boster) or Foxp3 (#bs‐10211R, Bioss) immunohistochemical staining. Ten areas were randomly selected from each group of three mice, and the area infiltrated by inflammatory factors was calculated using ImageJ software.

### Therapeutic Efficacy Assessment—Flow Cytometry

Regionally draining cervical lymph nodes (CLN) were harvested at the end of the experiment and single‐cell suspensions were prepared. The single cell suspension was stimulated with Cell Activation Cocktail (#423303, BioLegend) for 4 h at 37 °C. Then, cells were fixed, permeabilized, and stained with antibodies. Cell surface proteins were stained with FITC‐anti‐CD4 antibody (#100509, BioLegend) for 20 min at 4 °C, and intracellular cytokines were stained with PE/Cyanine7‐anti‐IFN‐γ (#505825, BioLegend) for Th1 cells, APC‐anti‐IL‐17A antibody (#506915; BioLegend) for Th17 cells, or PE‐antiFoxp3 antibody (#320007, BioLegend) for Tregs for 1 h at room temperature. Stained cells were collected on a flow cytometer (BD LSRFortessa, USA). Data were analyzed with FlowJo software.

### Therapeutic Efficacy Assessment—Luminex analysis

The inflammatory cytokines and chemokines of lacrimal glands were detected by a Luminex protein biochip testing system. Briefly, the lacrimal glands were harvested and homogenized in the lysate, then centrifuged and the supernatant was collected. The following cytokines in the supernatant were detected using the Bio‐Plex Pro Mouse Cytokine Grp (#M60009RDPD, Bio‐Rad) and Bio‐Plex 200 system (Luminex Corporation, Austin, TX, USA) as operating instructions: Eotaxin, G‐CSF, GM‐CSF, IFN‐γ, IL ‐1α, IL‐1β, IL‐2, IL‐3, IL‐4, IL‐5, IL‐6, IL‐10, IL‐12, IL‐13, IL‐17, MCP‐1, MIP‐1α, MIP‐1β, RANTES and TNF‐α.

### Statistical Analyses

Results were analyzed using GraphPad Prism 8 software. Differences between the two groups were compared using unpaired student *t*‐tests. For multiple comparisons, statistical significance was analyzed by one‐way analysis of variance (ANOVA), followed by Tukey's multiple comparisons test in case of equal SDs, and Games‐Howell's multiple comparisons test in case of non‐equal SDs. The level of statistical significance was set at *p <* 0.05. ^*^p < 0.05, ^**^p < 0.01, ^***^p < 0.001. All data were expressed as mean ± standard deviation (SD) unless otherwise indicated.

## Conflict of Interest

The authors declare no conflict of interest.

## Supporting information



Supporting Information

## Data Availability

The data that support the findings of this study are available from the corresponding author upon reasonable request.
