# Supplementary material for: ROS‐Responsive Microneedle Patches Enable Peri‐Lacrimal Gland Therapeutic Administration for Long‐Acting Therapy of Sjögren's Syndrome‐Related Dry Eye
Source: Adv Sci (Weinh). 2025 Jan 10;12(16):2409562. doi: 10.1002/advs.202409562 (PMC12021097; doi:10.1002/advs.202409562)
Supplement: Supplementary file 1 — Supporting Information [file ADVS-12-2409562-s001.docx]

Supporting Information

**ROS-responsive Microneedle Patches Enable Peri-lacrimal Gland Therapeutic Administration for Long-acting Therapy of Sjögren's Syndrome-related Dry Eye**

*Jingqing Mu, Xiangyu Ding, Yapeng Song, Baoyue Mi, Xiaolong Fang, Baihua Chen, Bin Yao, Xuguang Sun, Xiaoyong Yuan*, Shutao Guo*, Xia Hua**

J. Mu, B. Mi, X. Fang, Prof X. Hua

Aier Eye Hospital, Tianjin University, Fukang Road, Tianjin 300110, China

Email: [huaxia@aierchina.](mailto:huaxia@aierchina.)com

J. Mu, Prof. X. Hua

Changsha Aier Eye Hospital, Changsha, Hunan Province 410015, China

Aier Eye Institute, Changsha, Hunan 410009, China

X. Ding

Aier Academy of Ophthalmology, Central South University, Changsha, Hunan 410125, China

Y. Song, Prof. S. Guo

Key Laboratory of Functional Polymer Materials of Ministry of Education, State Key Laboratory of Medicinal Chemical Biology, Frontiers Science Center for New Organic Matter, College of Chemistry, Nankai University, Tianjin 300071, China

Email: [stguo@nankai.edu.cn](mailto:stguo@nankai.edu.cn)

Prof. B. Chen

Department of Ophthalmology, The Second Xiangya Hospital, Central South University, Hunan 410011, China

Prof. B. Yao

Academy of Medical Engineering&Translational Medicine, Medical College, Tianjin University, Tianjin 300072, China

Prof. X. Sun

Beijing Institute of Ophthalmology, Beijing Tongren Eye Center, Beijing Key Laboratory of Ophthalmology and Visual Sciences, Beijing Tongren Hospital, Capital Medical University, Beijing 100005, China

Prof. X. Yuan

Tianjin Key Laboratory of Ophthalmology and Visual Science, Tianjin Eye Institute, Tianjin Eye Hospital, Tianjin 300020, China

Email: [yxy@oio.cn](mailto:yxy@oio.cn)

Jingqing Mu, Xiangyu Ding contribute equally to this work.

**MATERIALS AND METHODS**

**Materials**

## N-Vinyl-2-Pyrrolidinone (NVP) and 3- (Acrylamido) phenylboronic acid (3APBA) were purchased from Bidepharm Co., Ltd (Shanghai, China). Irgacure 2959 was purchased from Mackin Biotechnology Co., Ltd (Shanghai, China). Cyclosporin A (CsA), (-)-Epigallocatechin gallate (EGCG), rhodamine B, fluorescein isothiocyanate (FITC), Cyclosporin D, ethyl gallate, and Folin&Ciocalteu phenol reagent was purchased from Yuanye Biotechnology Co., Ltd. (Shanghai, China). Alizarin Red S (ARS) was obtained from Solarbio Technology Co., Ltd (Beijing, China). Poly(N-isopropylacrylamide-co-butylacrylate) (PNIPAM-B, butylacrylate 12 mol%, average M_w_ 30,000) was obtained from Sigma-Aldrich (Missouri, USA). Polyvinyl alcohol (PVA) was obtained from EFL Co., Ltd (Suzhou, China). 2,2-Diphenyl-1-picrylhydrazyl (DPPH) and Calcein-AM and propidium iodide were obtained from Yeasen Biotechnology Co., Ltd. (Shanghai, China). DCFH-DA and Cy5 were purchased from Meilun Biotechnology Co., Ltd. (Dalian, China).

**Instrumentation**

The optical and fluorescent images of microneedles (MNs) were captured on a stereo microscope (Leica, Germany). The SEM images of MNs were captured on a JSM-7800F SEM (JEOL Company Ltd., Japan) with an accelerating voltage of 30 kV. Fluorescence spectrums were detected by an F-7000 fluorospectro photometer (Hitachi, Japan). The absorbance was measured using the microplate reader (Tecan, Austria). The fluorescent images were observed by a DMi8S fluorescent microscope (Leica, Germany). The fluorescent images of mice were obtained using the IVIS spectrum (PerkinElmer, USA).

**ARS fluorescence assay**

Briefly, 10 μM 3APBA and 10 μM ARS were dissolved in NVP solutions. The fluorescence intensity of the mixtures at 469 nm was measured within 2 h using the fluorospectro photometer. Subsequently, the ARS fluorescence assay was further performed to evaluate the binding efficiency between APBA and EGCG. 3APBA (10 μM), ARS (10 μM), and various amounts of EGCG (0, 50, 100, 500 μM) were dissolved in NVP solution for 3 h. The fluorescence signal of the mixtures was recorded by the fluorospectro photometer.

**Skin insertion of MN patches**

After hair removal from the dorsal skin of the mice, they were euthanized, and their dorsal skin was immediately cut off and placed on a clean glass slide. The fascia of the back skin of the mice was wiped off with cotton swabs dipped in PBS. The MN patch was left on the surface of the skin and then manually pressed for 30 s. After removal, 5% trypan blue was used for staining. After 1 min, the dye was removed and this process was repeated 3 times. The skin penetration property of MN was observed through a stereomicroscope. To further observe the penetration of the MN, the skin sample was immersed in 4% paraformaldehyde, then fixed in paraffin, cut into 5 μm sections, and stained with H&E. The morphology of the penetration holes was captured using a panoramic scanner (3DHISTECH, Hungary).

**The ROS responsive abilities of MN matrix**

To evaluate the drug release behavior of the MN, the samples were incubated in 2 mL phosphate buffer (pH 7.4) containing 0.01% (*v*/*v*) Tween 80 at 37 °C. At predetermined time points, 100 µl of supernatant was removed and an equal volume of fresh buffer was added. The released amount of CsA was determined by high-performance liquid chromatography. CsA was separated on a Poroshell 120 EC-C_18_ column (100 × 4.6 mm, 2.7 μm) at 60 °C with a mobile phase of acetonitrile containing 0.1% phosphoric acid (A) and water (B) according to the gradient elution procedure as shown in **Table S1**. The flow rate was 1.0 mL/min, and CsA was detected at wavelength of 210 nm. To assess the ROS sensitivity of the MN matrix, the samples were incubated in 2 mL phosphate buffer (pH 7.4, containing 0.01% Tween 80) with 0, 0.2, and 0.5 mM H_2_O_2_ at 37 °C. The released CsA was measured using the same method described above. The released EGCG was measured by the Folin-Ciocalteu method.^[1]^ Briefly, 40 μL of the sample was mixed with 40 μL of Folin & Ciocalteu phenol reagent. After 3 minutes, 120 μL of 10.75 wt% sodium carbonate was added, followed by incubation in the dark for 3 h. The absorbance of the final solution was measured at 760 nm, and the EGCG content was calibrated against an EGCG standard curve.

**Cell viability assay**

NIH3T3 cells were cultured in DMEM containing 10% FBS and 1% penicillin/streptomycin at 37 °C in a humidified environment with 5% CO2. HCEC cells were cultured in DMEM/F12 containing 5% FBS, insulin (5 μg/mL), hEGF (10 ng/mL), and 1% penicillin/streptomycin. The MN matrix was completely dissolved in a small amount of ethanol as a storage solution. The cytotoxicity of the formulations was assessed by the Cell Counting Kit-8 (CCK-8) assay. Cells were inoculated into 96-well plates (1×10^4^ cells per well) at 37 °C for 24 h. The medium was then removed and replaced with fresh medium containing serial concentrations of E-MN or CE-MN. After a further 24 h of incubation, the medium was replaced with a fresh DMEM medium containing 10% CCK-8 and incubated for 1.5 h. The absorbance of each well was analyzed by a microplate reader at 450 nm. Cell viability was calculated by the following equation:

$$\text{Cell viability\%=}\frac{\text{Absorbance}_{\text{sample}}\text{-}\text{Absorbance}_{\text{blank}}}{\text{Absorbance}_{\text{control}}\text{-}\text{Absorbance}_{\text{blank}}}\text{×100\%}$$

Besides, live/dead fluorescence imaging of treated cells was also performed using Calcein-AM and propidium iodide staining. Briefly, the cells were seeded in a 24-well plate (3×10^4^ cells per well) and incubated for 24 h. Then, the culture medium was replaced with a medium containing 200 μg/mL E-MN or CE-MN. After incubation for 24 h, all groups were stained by Calcein-AM and propidium iodide according to the instructions and visualized under a fluorescence microscope.

***In vivo* fluorescence imaging**

An Animal Imaging system was utilized to assess the smart release behaviors of MNs *in vivo*. Each lacrimal gland of the mouse was injected with 10 μL Canavalin A (ConA, 10 mg/mL) to induce the Sjögren's syndrome-related dry eye (SSDE) model. Two days later, Cy5-labelled CE-MNs were applied to the skin over the lacrimal gland of the SSDE mouse model or the healthy mouse. An IVIS Animal Imaging system (*λ*ex = 640 nm, *λ*em = 680 nm) was used to record the drug release behavior of the MNs at the predetermined time points.

**Detection of CsA and EGCG accumulation in lacrimal gland**

To assess the *in vivo* responsiveness of CE-MN to ROS and its subsequent drug release, healthy or SSDE mice were sacrificed at 24, 48, 72, and 96 h post-administration of CE-MN. The lacrimal glands were harvested to determine the distribution of CsA and EGCG. The lacrimal gland was homogenized in 20-fold of its volume with 50% aqueous methanol with 0.05 M zinc sulfate. The homogenate was then centrifuged at 5000 rpm for 10 min. For CsA extraction, an aliquot of 200 μL of the supernatant was vortex-mixed with 10 μL of internal standard solution (50 μg/mL, Cyclosporin D), and the mixture was extracted with ether (2 mL) by vortex-mixing for 5 min. After centrifugation at 12,000 rpm for 10 min, the clear supernatant was collected and dried using Eppendorf Concentrator (Eppendorf Co., GER). The residue was reconstituted with 100 μL of mobilephase, and 30 μL sample was injected for analysis. All samples were determined by a HPLC system. Separation was performed on a Poroshell 120 EC-C_18_ column (100 × 4.6 mm, 2.7 μm) at 60 °C with a mobile phase of acetonitrile containing 0.1% phosphoric acid/water (*v*/*v* = 75/25). The flow rate was 1.0 mL/min, and CsA and Cyclosporin D was detected at wavelength of 210 nm.

For EGCG extraction, an aliquot of 200 μL of the supernatant was vortex-mixed with 10 μL of internal standard solution (50 μg/mL, ethyl gallate) and 20 μL of preservation solution (0.2 % ascorbic acid and 0.005%Na_2_-EDTA), and the mixture was extracted with ethyl acetate (2 mL) by vortex-mixing for 5 min. After centrifugation at 12,000 rpm for 10 min, the clear supernatant was collected and dried using Eppendorf Concentrator (Eppendorf Co., GER). The residue was reconstituted with 100 μL of mobilephase, and 30 μL sample was injected for analysis. All samples were determined by a HPLC system. Separation was performed on a Poroshell 120 EC-C_18_ column (100 × 4.6 mm, 2.7 μm) at 30 °C with a mobile phase of acetonitrile containing 0.1% phosphoric acid/water (*v*/*v* = 15/85). The flow rate was 1.0 mL/min, and EGCG and ethyl gallate was detected at wavelength of 275 nm.

**The H2O2-scavenging efficiency of MNs**

The radical scavenging activity of MNs was evaluated using a DPPH scavenging assay and reactive oxygen species assay. First, the DPPH scavenging capacity was evaluated. E-MN or CE-MN was dissolved in ethanol solution to obtain a mixture from 10 μg/mL to 400 μg/mL; then the solution was mixed in equal volumes with an ethanol solution containing 200 μM DPPH and incubated for 30 minutes in the dark at room temperature. Pure EGCG with an equivalent content of CE-MN was also treated. The absorbance of the final solution was detected at 517 nm. The percentage of radical scavenging was calculated according to the following formula: DPPH scavenging % = (A_blank_- A_sample_ / A_blank_) × 100, where ABlank and AHydrogel are the absorbance of the blank (DPPH + ethanol) and the absorbance of the MN (DPPH + ethanol + sample) at 517 nm, respectively.

To evaluate the intracellular ROS scavenging ability, NIT3T3 cells (2×10^5^ cells/well) were seeded in 24-well plates and incubated for 12 h. Subsequently, the cells were first treated with H_2_O_2_ (3 μM) for 0.5 h followed by washed with PBS three times. Then, the cells were disposed with medium containing E-MN, CE-MN or EGCG (calculated as CE-MN equivalent concentration: 100 μg/mL) for 12 h. After that, DCFH-DA (20 μM) was added and incubated for 30 min. Cells treated with H_2_O_2_ alone served as the positive control, while untreated cells were used as the negative control. Afterward, the cells were imaged using a fluorescence microscope.

***In vitro* anti-inflammatory activities of MNs**

Raw 264.7 macrophages were seeded in 24-well plates at a density of 1×10^5^ cells per well. After 24 h of incubation, the medium was discarded, and fresh medium containing E-MN, CE-MN, CsA, or EGCG (calculated as CE-MN equivalent concentration: 100 μg/mL) was added for 3 h of preincubation. Then, lipopolysaccharide (LPS) was added to each well (final concentration: 1 μg/mL). After coincubation for 21 h, the supernatants were collected and centrifuged at 12000 rpm for 10 min. Cells treated with LPS alone were used as the positive control, while untreated cells were used as the negative control. The concentrations of TNF-α and IL-6 in the supernatants were quantified by corresponding enzyme-linked immunosorbent assay (ELISA) kits (#EK282 and #EK206, Multisciences). The concentrations of NO in the supernatants were measured by Griess reagents (#G9440, Solarbio). A similar method was used to assess the efficacy of the CE-MN as a therapeutic strategy. Macrophages were firstly stimulated with LPS (1 μg/mL) for 1 h. Then, different formulations (calculated as CE-MN equivalent concentration: 100 μg/mL) were added to the culture medium. After 24 h, samples were collected and detected.

To further validate the anti-inflammatory effect of MNs, Western blotting analysis was performed to detect the protein expression of iNOS and COX2 in treated cells. Cells were treated as described above, then total protein lysate was extracted from the cells and determined using a BCA protein assay kit (#P0011, Beyotime) and analyzed by SDS-polyacrylamide gel electrophoresis, followed by transfer to a polyvinylidene difluoride membrane. The anti-iNOS (1:1000, #340668) and anti-COX2 (1:1000, #R23971) antibodies were purchased from Zen-bioscience. The horseradish peroxidase (HRP)-conjugated anti-rabbit IgG (#7074) and anti-mouse IgG (#7076) were purchased from Cell Signaling Technology (USA). Densitometric scanning normalized against β-actin was used to assess relative protein concentration. Blots were imaged by an Imaging system (Tanon5200, Shanghai, China).

**The study of insertion position of CE-MN patches post-administration**

To investigate the location and fate of MN tips in the peri-lacrimal gland of SSDE mice, the rhodamine B-labeled CE-MN was applied by peri-lacrimal gland administration. The periocular tissue histological images at different time points after MN application (1 and 48 h) were captured by a microscope.

**Cy5 accumulation in lacrimal gland**

The SSDE model was constructed using ConA injection. After 24 h, the Eye drop group received eye drops containing Cy5 twice a day for two days. The CE-MN group was coated with MN patches containing Cy5 (the amount of Cy5 was the same as the amount in the eye drops administered for two days). All mice were sacrificed and lacrimal glands were collected for imaging using IVIS Animal Imaging system (*λ*ex = 640 nm, *λ*em = 680 nm) at 12, 24, 48 h post treatment.

**Supplementary Figures**


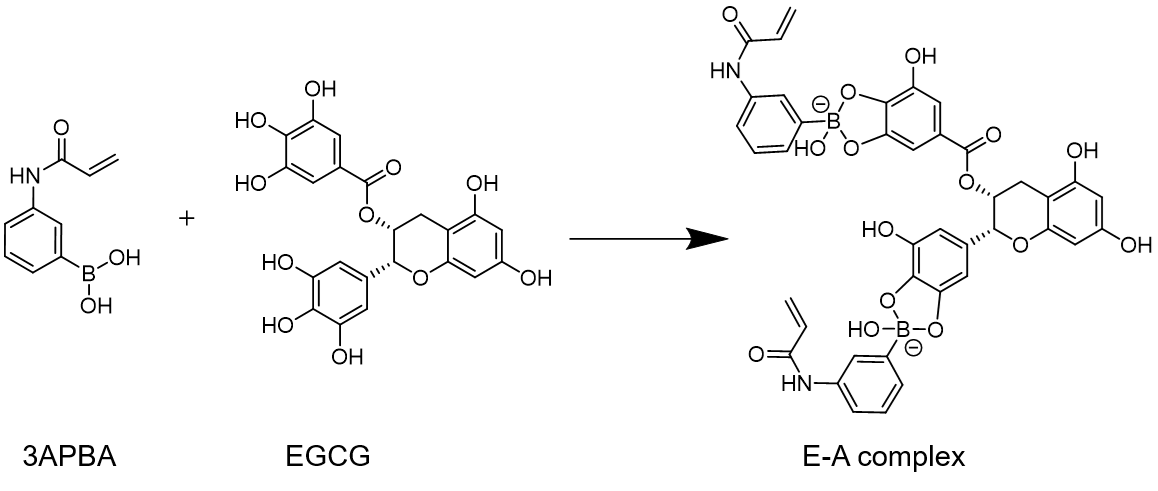


**Figure S1**. The synthesis route of E-A complex.


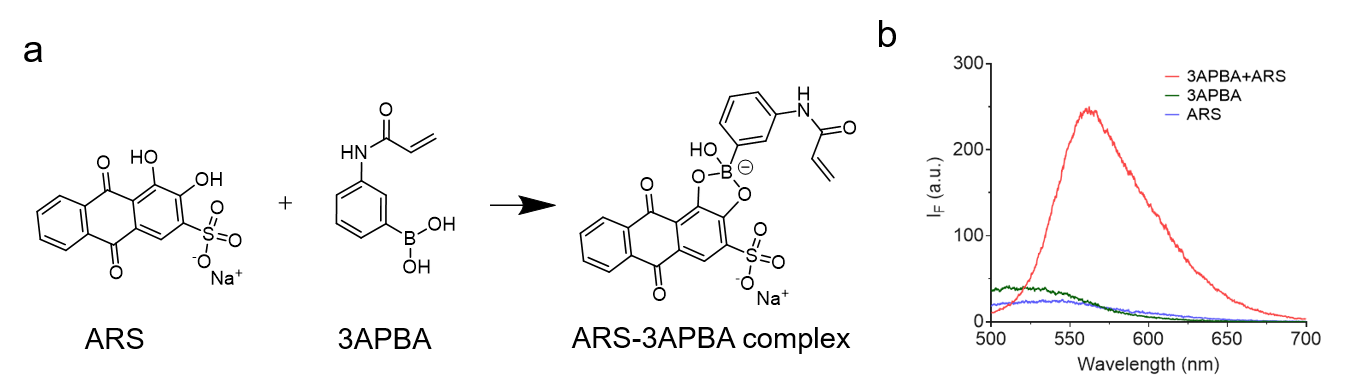


**F****igure S2.** a) The synthesis route of ARS-3APBA complex. b) Fluorescence spectra of ARS (10 μM) incubated with 3APBA (10 μM) for 2 h in NVP solution. Excitation: 469 nm.


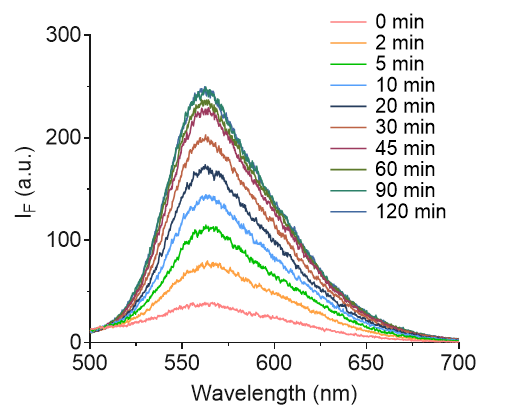


**Figure S3.** Fluorescence spectra of ARS (10 μM) incubated with 3APBA (10 μM) in NVP solution at different times. Excitation: 469 nm.


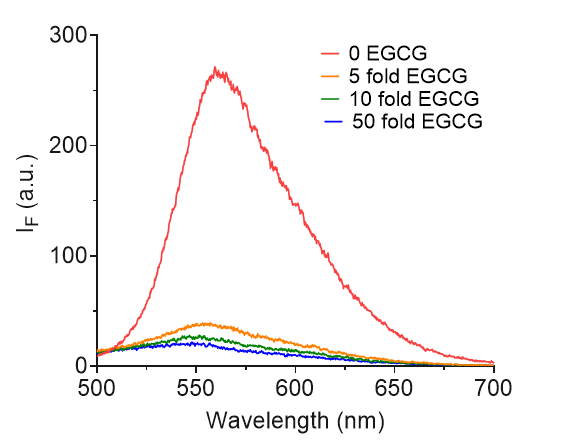


**Figure S4.** Fluorescence spectra of ARS (10 μM) incubated with 3APBA (10 μM) and different ratios of EGCG (0, 50, 100, 500 μM) in NVP for 3 h. Excitation: 469 nm.


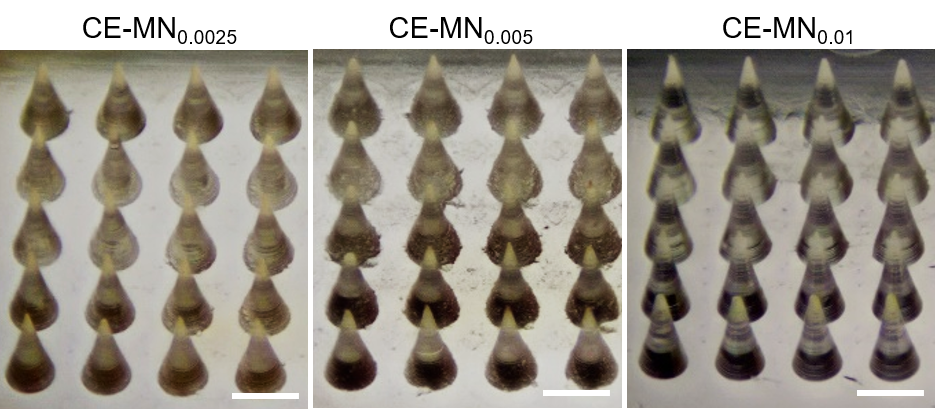


**Figure S5.** Typical digital photographs of three kinds of CE-MN. scale bar: 400 μm.


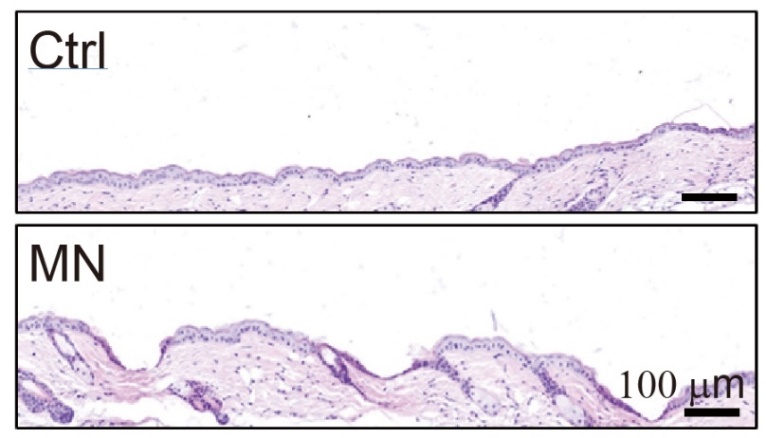


**Figure S6.** Typical H&E images of mice skin after MN pressing.


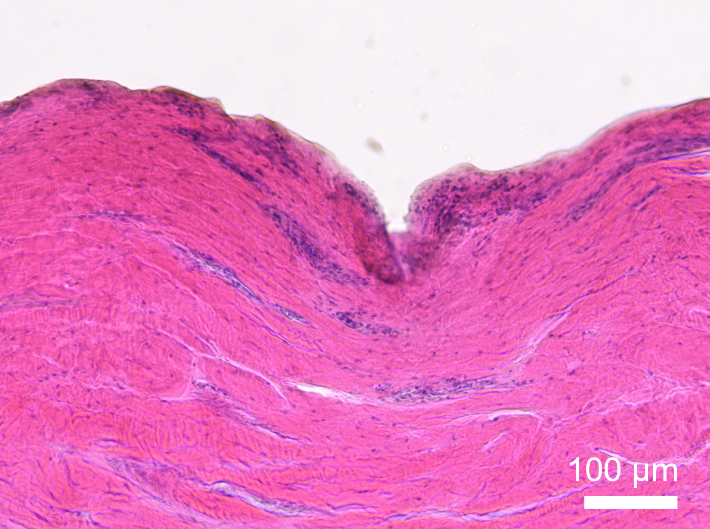


**Figure S7.** Typical H&E images of porcine skin after MN pressing.


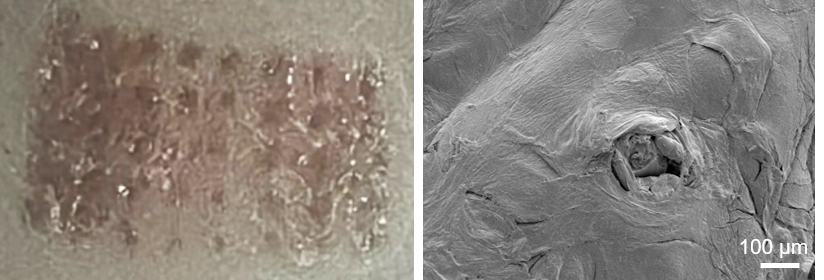


**Figure S8.** Digital photograph (left) and SEM image (right) of CE-MN patch after manually pressing in the skin of BALB/c mice. The patch has been cooled (∼10 °C) for 10 min.


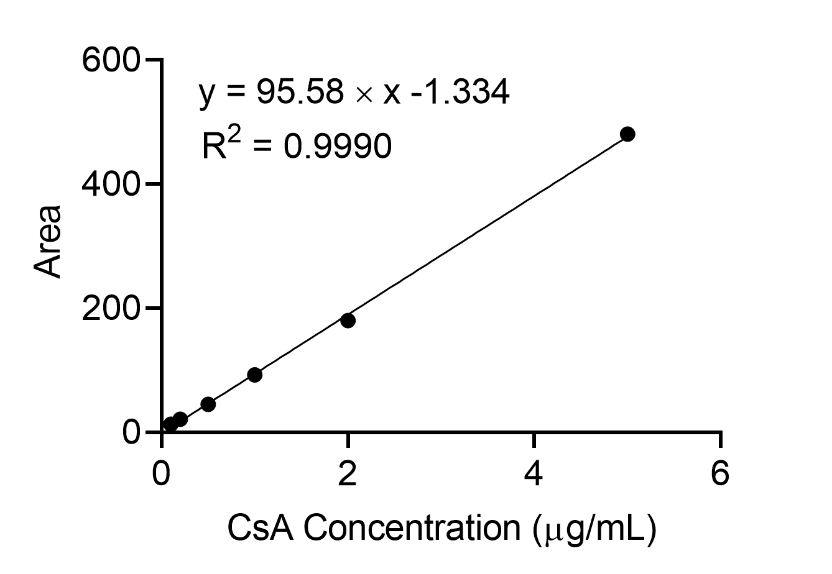


**Figure S9.** Standard curve for CsA determined by HPLC.


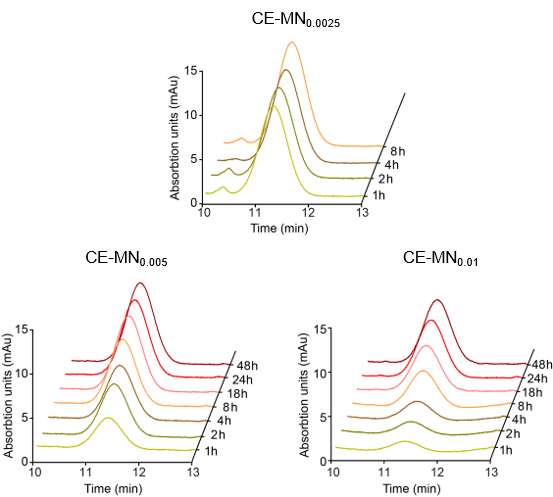


**Figure S10.** HPLC release chromatograms of different kinds of CE-MN at pH 7.4. Retention time was 11.35 min for CsA.


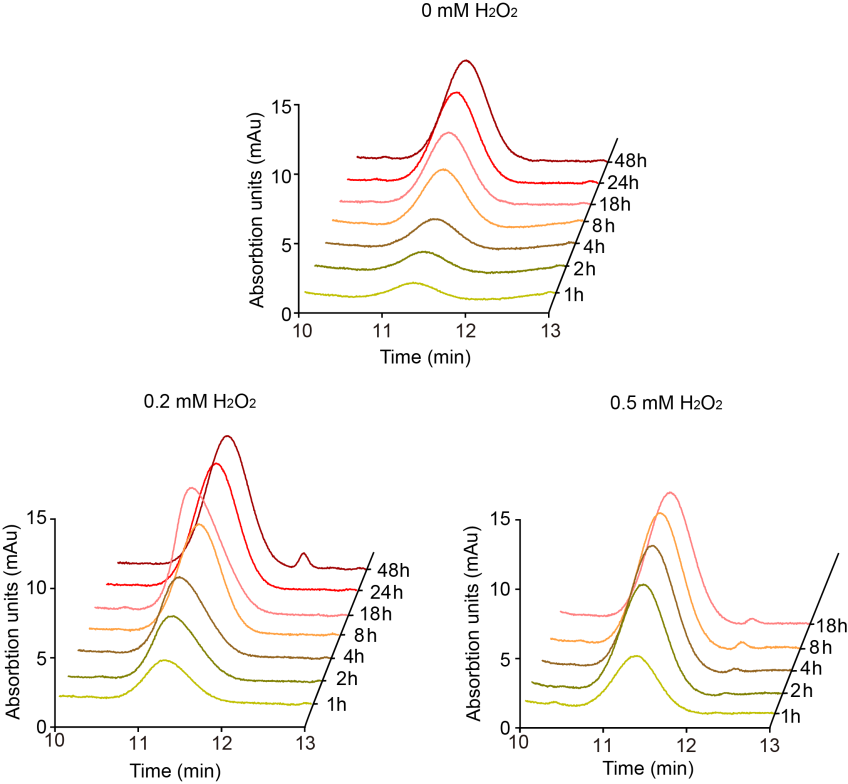


**Figure S11.** HPLC release chromatograms of CE-MN at different concentrations of H_2_O_2_. Retention time was 11.35 min for CsA.


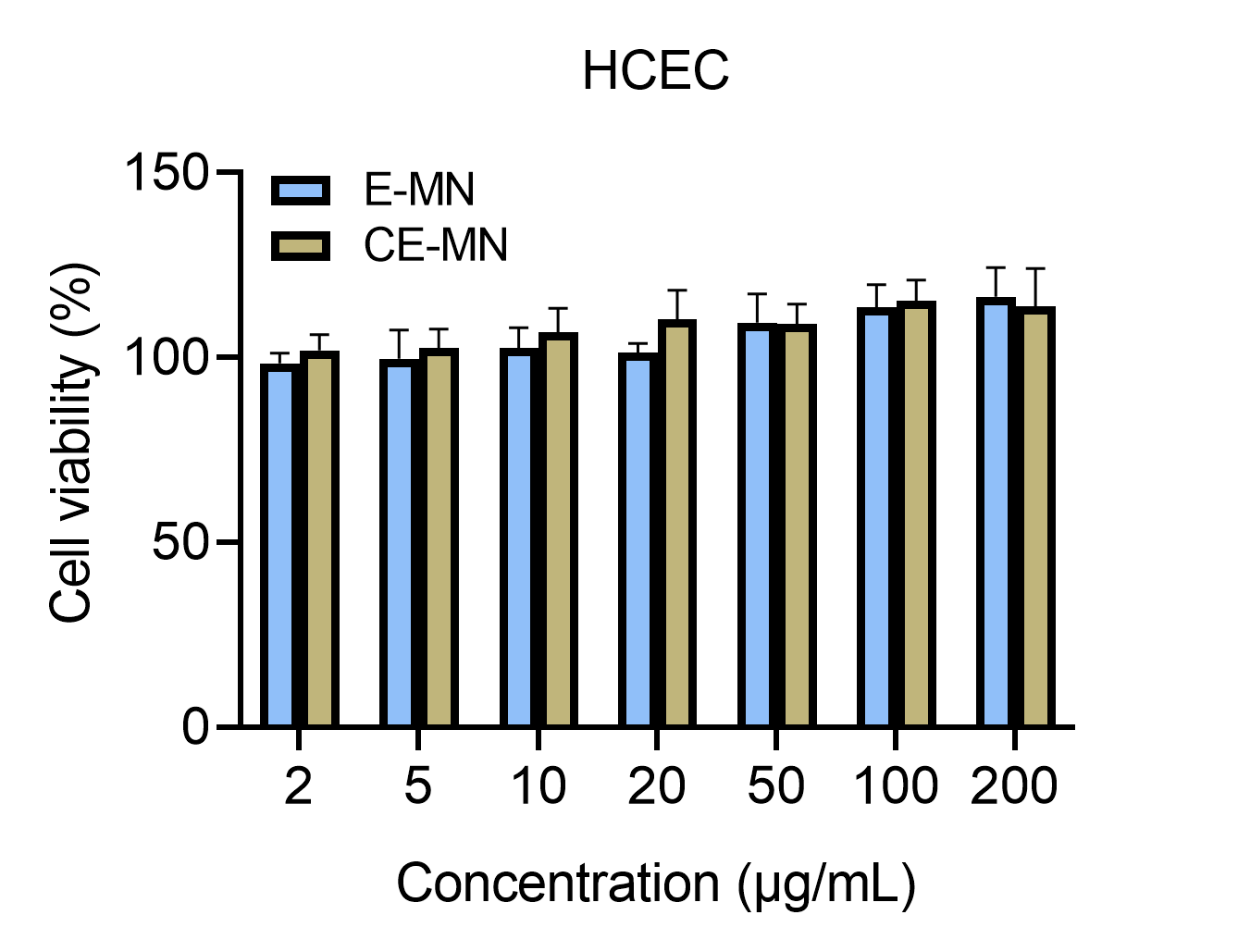


**Figure S12.** Cell viability of HCEC cells after co-culturing with E-MN and CE-MN for 24 h. All data are presented as mean ± SD (*n* = 3).


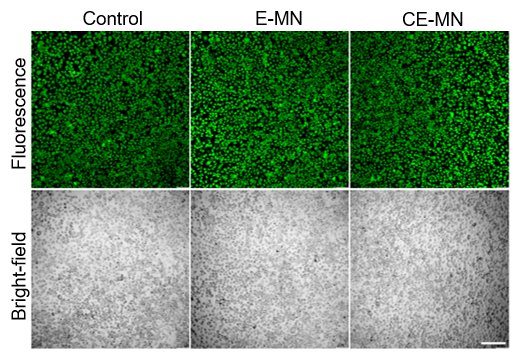


**Figure S13.** Live (green)/dead (red) staining fluorescence images of HCEC cells after co-culturing with E-MN and CE-MN for 24 h. scale bar: 200 μm.


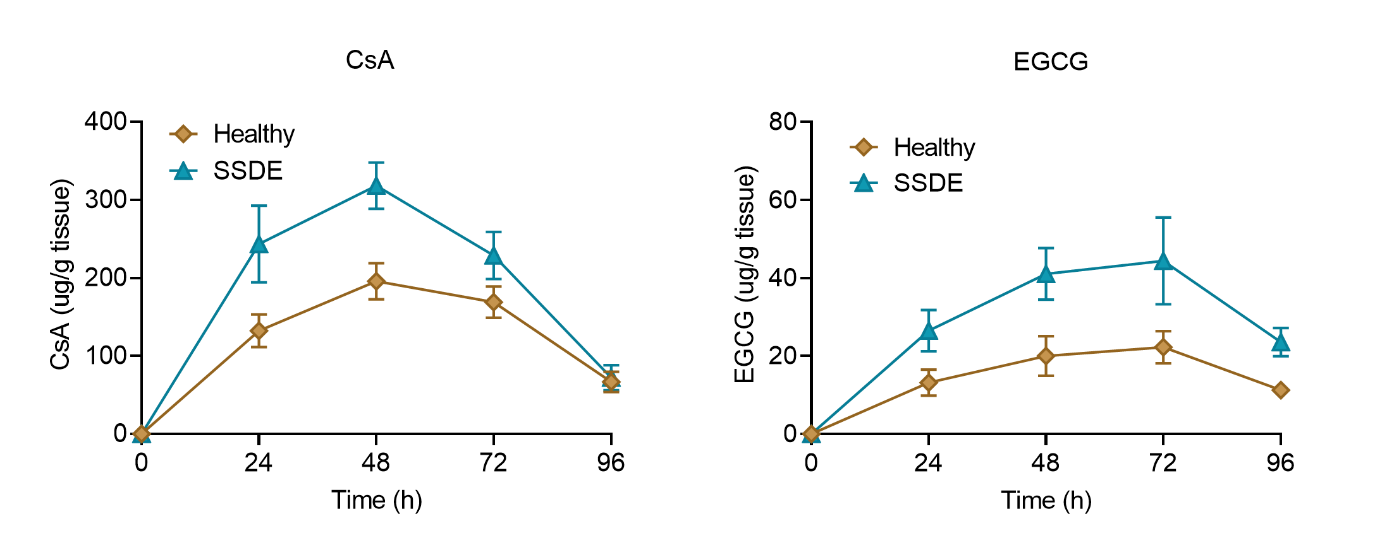


**Figure S14.** The concentration of CsA and EGCG in the lacrimal gland at different time points after administration of CE-MN patch on the SSDE mice or healthy mice (*n* = 3, mean ± SD).


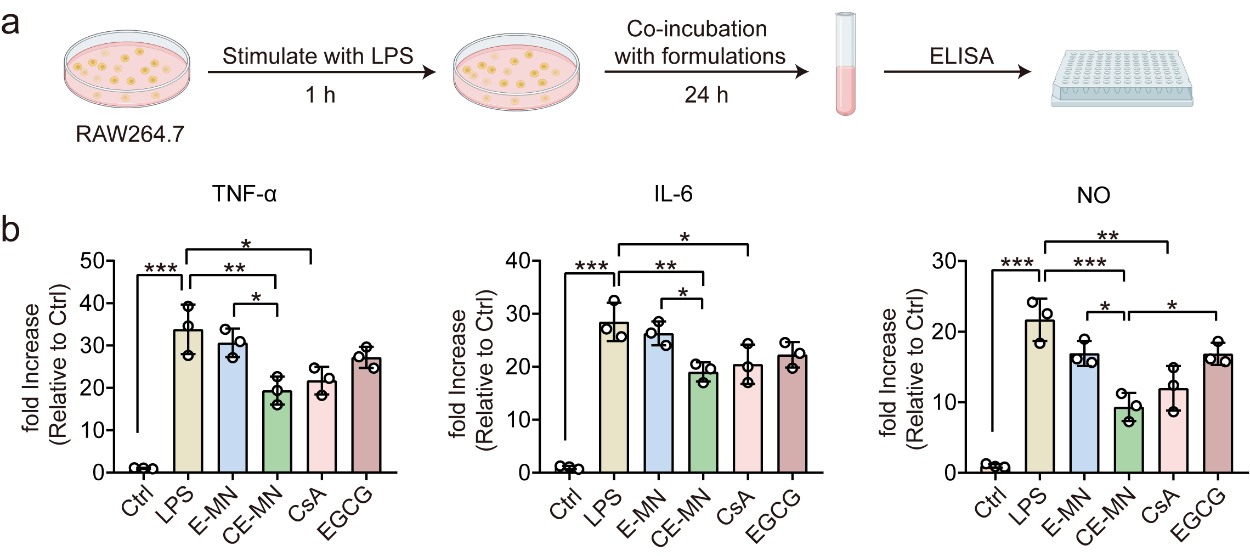


**Figure S15.** a) Scheme of the experimental procedures to access the inflammation level in RAW264.7 cells. Macrophages were stimulated with LPS (1 μg/mL) for 1 h, then various formulations were added, and samples were incubated for an additional 24 h prior to detection. b) Effects of CE-MN and other formulations on TNF-α, IL-6, and NO production in RAW264.7 macrophages stimulated with LPS (*n* = 3, mean ± SD).


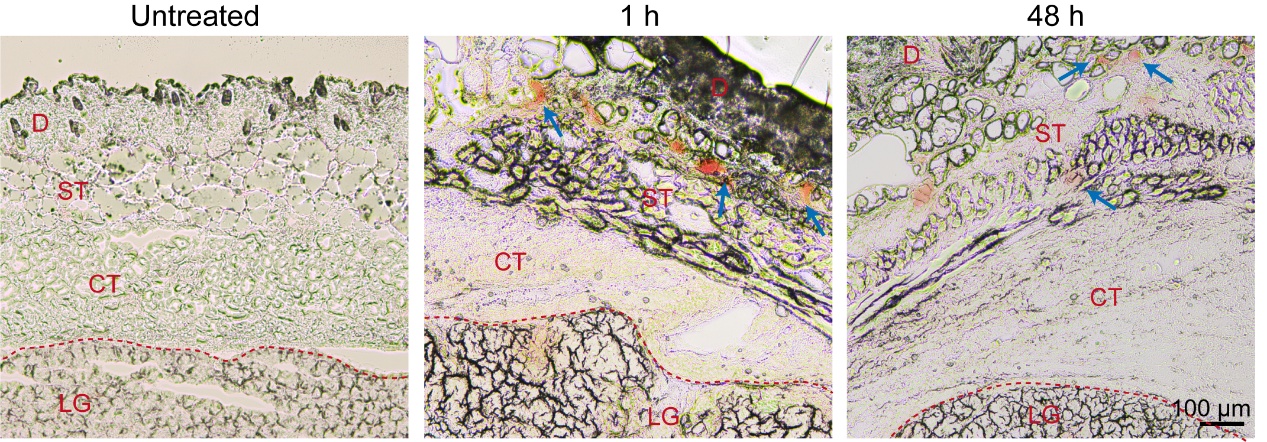


**Figure S16.** The frozen sections of periocular tissue in SSDE mice untreated or treated with Rhodamine B-loaded MN for 1 or 48 h. Microneedle matrix are the red region located in the subcutaneous tissue highlighted with blue arrows. The red dashed line represents the edge of the lacrimal gland. D, dermis. ST, subcutaneous tissue. CT, connective tissue. LG, lacrimal gland.


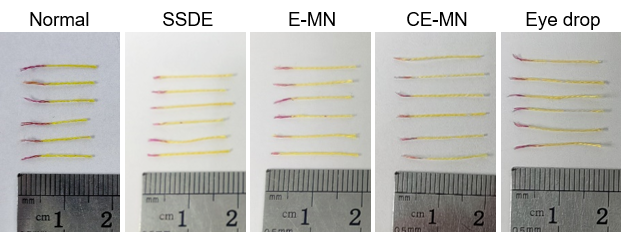


**Figure S17.** Wetted phenol red thread lengths for tear production evaluation after various treatments on the 7th day.


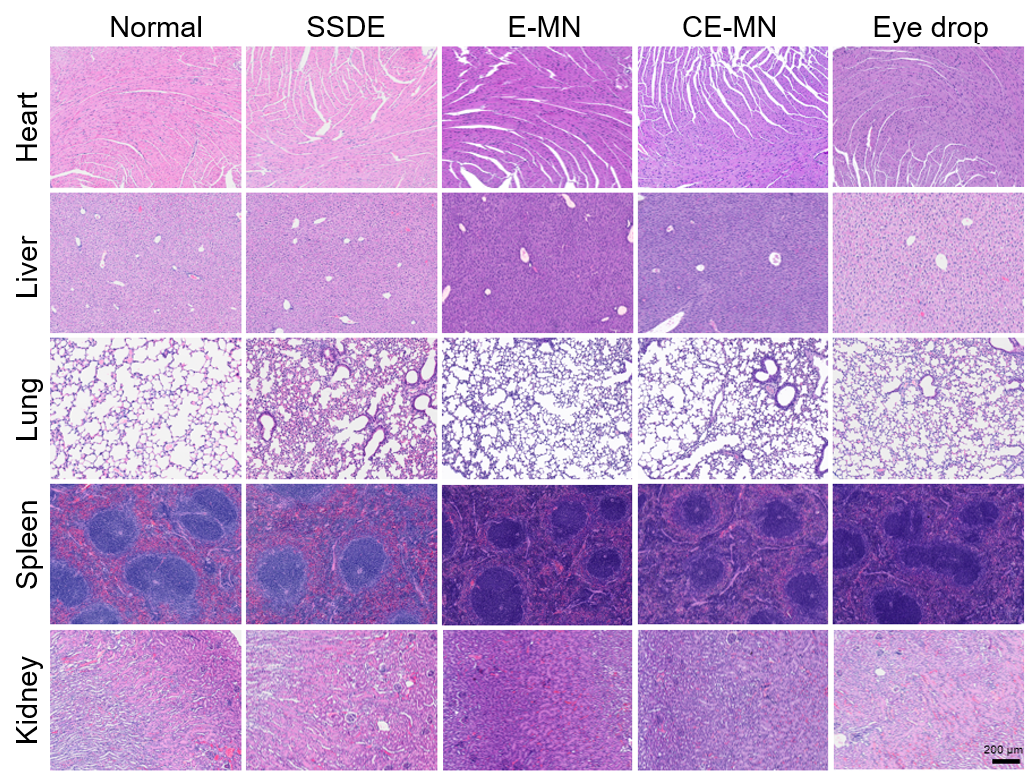


**Figure S18.** H&E staining of organs from mice treated with various formulations on the 7th day.

**Table S1.** The [gradient elution](https://www.sciencedirect.com/topics/chemistry/gradient-elution) used for HPLC determination of CsA.

| Time (min) | A(%) | B(%) |
| --- | --- | --- |
| 0.00 | 60 | 40 |
| 12.00 | 80 | 20 |
| 15.00 | 80 | 20 |
| 15.01 | 60 | 40 |
| 18.00 | 60 | 40 |

**References**

[1] B. Kim, S.-H. Kim, K. Kim, Y.-H. An, K.-H. So, B.-G. Kim, N. Hwang, *Mater. Today Bio* **2020**, *8*, 100079.
